# Effects of proprioceptive exercise for knee osteoarthritis: a systematic review and meta-analysis

**DOI:** 10.3389/fresc.2025.1596966

**Published:** 2025-06-24

**Authors:** Yaoyu Lin, Debiao Yu, Xiaoting Chen, Peng Chen, Nan Chen, Bin Shao, Qiuxiang Lin, Fuchun Wu

**Affiliations:** ^1^ College of Rehabilitation Medicine, Fujian University of Traditional Chinese Medicine, Fuzhou, China; ^2^ Provincial Clinical Medical College, Fujian Medical University, Fuzhou, China; ^3^ Rehabilitation Medicine Center, Fujian Provincial Hospital, Fuzhou, China; ^4^ Rehabilitation Medicine Center, Fuzhou University Affiliated Provincial Hospital, Fuzhou, China; ^5^ College of Acupuncture and Tuina, Fujian University of Traditional Chinese Medicine, Fuzhou, China; ^6^ Department of Rehabilitation Medicine, Quanzhou First Hospital Affiliated to Fujian Medical University, Quanzhou, China

**Keywords:** knee osteoarthritis, proprioceptive exercise, pain, balance function, rehabilitation, meta-analysis

## Abstract

**Background and objective:**

Despite the extensive utilization of proprioceptive exercise in the management of knee osteoarthritis (KOA), the therapeutic efficacy of this approach remains inconclusive. The present study sought to systematically evaluate the effects of proprioceptive exercise on symptoms and functional outcomes in patients with KOA, with a particular focus on balance performance.

**Methods:**

Following PRISMA guidelines, a comprehensive search was conducted across six electronic databases from the establishment of the database to January 21, 2025. The inclusion criteria were randomized controlled trials investigating proprioceptive exercise interventions for KOA. The primary outcome measures encompassed balance function assessment (Timed Up and Go test), Western Ontario and McMaster Universities Osteoarthritis Index (WOMAC) total score and its pain, stiffness, and function subscales, and pain intensity (Numerical Rating Scale and Visual Analog Scale). Subgroup analyses were stratified by intervention duration (≤8 weeks vs. >8 weeks).

**Results:**

A comprehensive analysis of 22 randomized controlled trials revealed that proprioceptive exercise significantly improved performance of the Timed Up and Go test [MD = 1.53, 95% CI (1.09, 1.97), *I*^2^ = 0%, *P* < 0.00001]. Additionally, a significant improvement in WOMAC-total scores was observed [MD = 3.37, 95% CI (1.58, 5.16), *I*^2^ = 44%, *P* = 0.0002]. However, individual WOMAC subscales for pain (*P* = 0.11, *I*^2^ = 85%), stiffness (*P* = 0.97, *I*^2^ = 0%), and function (*P* = 0.16, *I*^2^ = 86%) showed no significant improvements. For pain assessment, Numerical Rating Scale scores showed a significant improvement [MD = 0.85, 95% CI (0.56, 1.15), *I*^2^ = 46%, *P* < 0.00001]. Notably, Visual Analog Scale scores exhibited a significant reduction, but only in the short-term intervention subgroup (≤8 weeks) [MD = 0.27, 95% CI (0.11, 0.42), *I*^2^ = 0%, *P* = 0.0008], whereas longer interventions (>8 weeks) showed no significant benefit [MD = −0.49, 95% CI (−1.10, 0.11), *I*^2^ = 0%, *P* = 0.11].

**Conclusion:**

Based on low-certainty evidence, proprioceptive exercise has been demonstrated to be efficacious in improving balance function and overall clinical status in patients with KOA. Optimal benefits have been observed during short-term intervention periods.

## 1 Introduction

Knee osteoarthritis (KOA) is a common degenerative joint disease (1), with global epidemiological data indicating a prevalence of approximately 16% (2), which continues to increase with the aging population. Research has indicated that patients with KOA demonstrate a considerably elevated risk of falling, with approximately 50%–60% of patients with KOA over the age of 60 reporting at least one fall per year (3). This increased risk not only compromises quality of life but also imposes substantial economic burdens on patients, families, and healthcare systems (4, 5).

A substantial body of research has identified a strong correlation between falls and balance dysfunction (6, 7). Patients with KOA suffer from proprioceptive deficits due to structural changes in the joints and decreased mechanoreceptor function, which means that the body's ability to sense its own position, movement, and strength is impaired (8–10). The proprioceptive system relies primarily on mechanoreceptors located in joints, muscles, and ligaments to maintain normal motor control by transmitting information about joint angles, muscle tension, and speed of movement (11). As KOA progresses, impaired proprioceptive function results in a significant reduction in the accuracy of joint position and motion perception, which in turn weakens the effectiveness of the joint stability control system. When proprioceptive inputs are insufficient or inaccurate, the central nervous system is unable to accurately assess the body's position and motion status in space, resulting in temporal delays and inappropriate magnitude of compensatory postural adjustments. This condition manifests specifically as a decrease in anticipatory postural adjustment and a decrease in the efficiency of reactive balance control, and the patient walks with abnormalities such as shortened stride length, gait asymmetry, and increased gait variability, and the risk of falling is significantly increased, especially in complex environments such as turning corners, crossing an obstacle, or coping with uneven surfaces (12, 13). In summary, impaired proprioception weakens the function of the stability control system and ultimately increases the risk of falling.

Patients with KOA suffer from proprioceptive deficits due to structural changes in the joints and decreased mechanoreceptor function, which means that the body's ability to sense its own position, movement, and strength is impaired. The proprioceptive system relies primarily on mechanoreceptors located in joints, muscles, and ligaments to maintain normal motor control by transmitting information about joint angles, muscle tension, and speed of movement. As KOA progresses, impaired proprioceptive function results in a significant reduction in the accuracy of joint position and motion perception, which in turn weakens the effectiveness of the joint stability control system. When proprioceptive inputs are insufficient or inaccurate, the central nervous system is unable to accurately assess the body's position and motion status in space, resulting in temporal delays and inappropriate magnitude of compensatory postural adjustments. This condition manifests specifically as a decrease in anticipatory postural adjustment and a decrease in the efficiency of reactive balance control, and the patient walks with abnormalities such as shortened stride length, gait asymmetry, and increased gait variability, and the risk of falling is significantly increased, especially in complex environments such as turning corners, crossing an obstacle, or coping with uneven surfaces. In summary, impaired proprioception weakens the function of the stability control system and ultimately increases the risk of falling.

Currently, the predominant clinical intervention for proprioceptive dysfunction is proprioceptive exercise (14), which enhances neuromuscular control by means of the stimulation of the mechanoreceptors of the joints and muscles, thereby improving joint position sense and balance. The training modality of proprioceptive exercise is versatile and can be performed in a number of ways. These include joint position perception training (15, 16) or training with the aid of equipment such as wobble boards (17, 18). However, there is considerable heterogeneity in the implementation protocols for proprioceptive exercise, and the current research is divided in its evaluation of the efficacy of this intervention. Some studies have found proprioceptive exercise to be effective in improving pain and function in patients (19–21), while others have shown that the effects may be limited (22, 23). The findings from previous meta-analyses also demonstrate significant heterogeneity, with the study by Jeong et al. indicating that the effect of proprioceptive training on improving pain and function was in the lower range of the smallest clinically significant difference (24), whereas the study by Smith et al. suggests that it may be advantageous in terms of functional improvement (25). In a recent study, Wang et al. conducted a meta-analysis and found that proprioceptive training significantly improved pain, stiffness, physical function and proprioception in patients with KOA (26). However, these previous meta-analyses had notable limitations. Jeong et al.'s review was restricted to only seven randomized controlled trials with a limited cumulative sample size of 558 participants, while Smith et al. included only seven studies with a total of 560 participants. Neither study adequately explored the sources of the high heterogeneity observed in their analyses, despite reporting substantial heterogeneity in several outcomes (*I*^2^ reaching 65% for pain and 86% for function in the study by Jeong et al, and up to 88% for function in the study by Smith et al). Furthermore, their primary focus remained on outcomes such as pain, joint function and proprioception, while balance function, a crucial clinical indicator in patients with KOA, was inadequately addressed.

Based on the limitations, we conducted this systematic review and meta-analysis to comprehensively evaluate the effects of proprioceptive exercise on knee joint symptoms and function in patients with KOA, especially attention to its role in improving balance ability. Additionally, given that previous studies have indicated that adaptive changes in the neuromuscular system typically occur within 4–8 weeks (27–29), intervention duration may be an important factor affecting therapeutic efficacy. Consequently, this study investigated the possible influence of intervention duration on clinical outcomes. Through these analyses, we aimed to provide more comprehensive evidence-based support for clinical practice.

## 2 Methods

We conducted this study following the Preferred Reporting Items for Systematic Reviews and Meta-Analyses (PRISMA) guidelines (30) and registered the protocol with PROSPERO (registration number: CRD42025642296).

### 2.1 Search strategy

A comprehensive literature search was conducted in six electronic databases, including PubMed, Embase, Cochrane Library, Web of Science, CINAHL Complete, and Scopus. All searches were performed from the establishment of the database to January 21, 2025. The search strategy employed a combination of medical subject headings (MeSH) and free terms, including “knee osteoarthritis”, “proprioception”, and “randomized controlled trial”. The search strategy employed for the PubMed database is shown in Table 1, and other databases used a similar search strategy, adapted to the characteristics of each database (Supplementary S1). Additionally, manual screening of reference lists from included studies and relevant systematic reviews was conducted to identify any potentially eligible studies that may have been missed during the electronic database search.

**Table 1:** The search strategy for PubMed.

Query	Search term
#1	"osteoarthritis, knee"[MeSH Terms]
#2	"Osteoarthritis"[MeSH Terms] AND “Knee"[All Fields]
#3	"osteoarthrit*"[Title/Abstract] AND “knee*"[All Fields]
#4	"knee osteoarthritis"[Title/Abstract] OR “Osteoarthritis of Knee"[Title/Abstract] OR “Osteoarthritis of Knees"[Title/Abstract] OR “Knee OA"[Title/Abstract] OR “gonarthrosis"[Title/Abstract]
#5	#1 OR #2 OR #3 OR #4
#6	"Proprioception"[MeSH Terms]
#7	"Kinesthesis"[MeSH Terms]
#8	"Postural Balance"[MeSH Terms]
#9	"propriocep*"[Title/Abstract] OR “kinesthes*"[Title/Abstract] OR “position sense"[Title/Abstract] OR “movement sense"[Title/Abstract] OR “velocity sense"[Title/Abstract] OR “force sense"[Title/Abstract] OR “sensorimotor training"[Title/Abstract] OR “balance training"[Title/Abstract] OR “postural control"[Title/Abstract] OR “neuromuscular training"[Title/Abstract] OR “stability training"[Title/Abstract] OR “kinesthetic training"[Title/Abstract] OR “core stability"[Title/Abstract]
#10	#6 OR #7 OR #8 OR #9
#11	"Randomized Controlled Trial"[Publication Type]
#12	"Controlled Clinical Trial"[Publication Type]
#13	"randomized"[Title/Abstract] OR “randomised"[Title/Abstract] OR “randomly"[Title/Abstract] OR “trial"[Title/Abstract] OR “groups"[Title/Abstract]
#14	#11 OR #12 OR #13
#15	#5 AND #10 AND #14

### 2.2 Eligibility criteria

Two investigators independently reviewed titles and abstracts and evaluated the full texts of potentially eligible studies according to predetermined inclusion and exclusion criteria. Any disagreements were resolved through discussion or consultation with a third investigator.

Inclusion criteria encompassed: (a) randomized controlled trials (RCT); (b) participants with clinical or radiographic diagnosis of KOA according to established criteria; (c) proprioceptive exercise interventions, including proprioceptive training, apparatus-assisted exercise (e.g., wobble boards, unstable platforms), balance training, neuromuscular training, or sensorimotor training. In this review, proprioceptive exercise was operationally defined as structured physical interventions designed to enhance joint position sense, neuromuscular control, and balance function through targeted stimulation of mechanoreceptors in joints and muscles. To qualify for inclusion, interventions needed to meet the following minimum criteria: intervention duration of at least 2 weeks; for short-term interventions (≤8 weeks), a frequency of at least once a week on average; for long-term interventions (>8 weeks), a frequency of at least once every two weeks on average; (d) comparator groups comprising either a blank control or other non-surgical treatment methods (intervention measures can be implemented in clinical, family, or collaborative environments, potentially involving personalized or group training under professional supervision); and (e) published by English.

Exclusion criteria encompassed: (a) research not using an RCT design; (b) irrelevant research content; (c) insufficient data reported; and (d) redundant publications (multiple reports of the same study with the same participant population).

### 2.3 Data extraction

Two investigators independently extracted data using a standardized form. The extracted information included: (a) basic study characteristics (authors, publication year, country/region); (b) sample demographics and group allocation details; (c) comparator intervention; (d) experimental intervention; (e) frequency and intervention duration; (f) blinding (Non-Blinded, single-blinded, double-blinded, or patrial blinding); and (g) outcome measurements, including: Timed Up and Go (TUG) test results measured in seconds, with lower values indicating better mobility; Western Ontario and McMaster Universities Osteoarthritis Index (WOMAC) total scores (ranging from 0 to 96 points, with lower scores indicating better outcomes) and its subscales including pain (0–20 points), stiffness (0–8 points), and physical function (0–68 points); and pain assessment scores using the Numerical Rating Scale (NRS, 0–10 points) and Visual Analog Scale (VAS, 0–10 cm), with higher scores indicating greater pain intensity.

### 2.4 Assessment of study quality

The methodological quality of all included studies was independently assessed by two investigators. The Cochrane risk-of-bias tool (ROB 2) (31, 32) was utilized to evaluate the randomization process, deviation from expected interventions, completeness of outcome data, measurement of outcomes, and selectivity in reporting outcomes. For each domain, specific signaling questions were answered as “yes”, “probably yes”, “no information”, “probably no”, or “no” based on the information provided in the studies. Following the ROB 2 algorithm, each study was ultimately classified as having “low risk”, “some concerns”, or “high risk” for each domain and overall. The detailed assessment criteria and decision-making process are provided in the Supplementary S2. The certainty of evidence for each outcome was evaluated using the Grading of Recommendations Assessment, Development and Evaluation (GRADE) approach, considering risk of bias, inconsistency, indirectness, imprecision, and publication bias. Any disagreements were resolved through discussion or consultation with a third investigator.

### 2.5 Data analysis

Statistical analyses were performed using RevMan 5.4 and Stata 17.0 software. For missing standard deviations, conversions were made using reported data. For continuous variables, mean differences (MD) and 95% confidence intervals (CI) were calculated. Positive values indicate favorable outcomes for the experimental group. Heterogeneity was assessed using the *I*^2^ statistic, with *I*^2^ ≤ 50% indicating no significant heterogeneity or low heterogeneity, suggesting possible common effect sizes, and thus a fixed-effects model was used, and *I*^2^ > 50% indicating high heterogeneity, suggesting possible different true effects and the need to account for between-study differences, and thus a random-effects model was used (33, 34). Separate meta-analyses were conducted for each outcome measure. Pre-specified subgroup analyses were performed for all outcome measures according to intervention duration (≤8 weeks vs. >8 weeks). We also conducted subgroup analyses by publication year, intervention type (single vs. combined), and study region (Asian vs. non-Asian) to explore sources of heterogeneity (the results of intervention type and study region are presented in the Supplementary Materials). Sensitivity analyses were conducted by removing individual studies one by one, with an effect estimate change greater than 20% after exclusion regarded as an unstable result (detailed data presented in Supplementary S3). Publication bias for each outcome measure was evaluated using Egger's test. Funnel plots were used to assess publication bias for outcome measures that included more than 10 studies. Statistical significance was set at *P* < 0.05.

## 3 Results

### 3.1 Study selection results and characteristics

**Figure 1:**
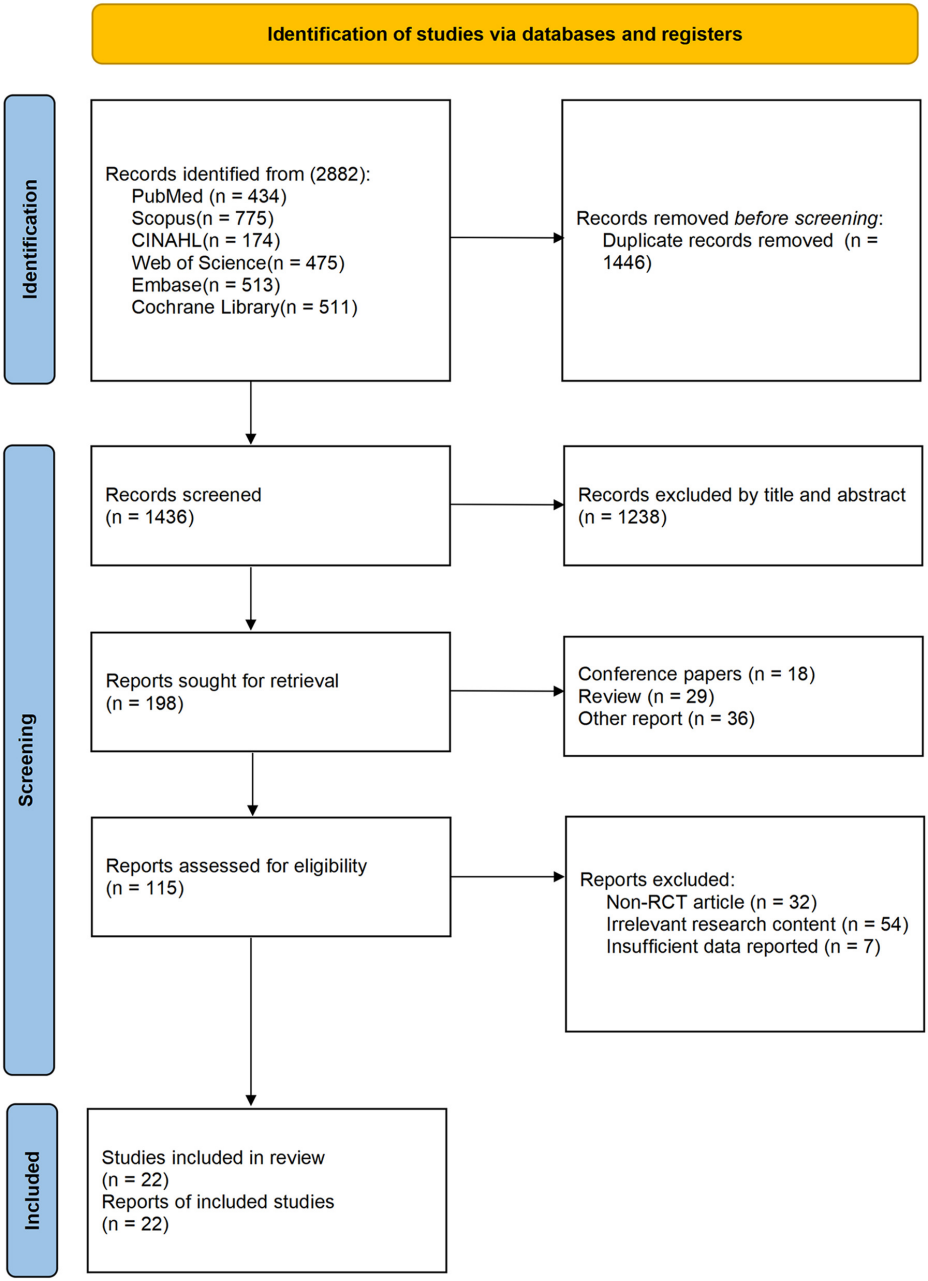
PRISMA study selection flowchart.

A comprehensive database search yielded 2,882 potentially relevant articles across six electronic databases, with 1,436 remaining after duplicate removal. Title and abstract screening identified 198 articles for full-text assessment. We subsequently excluded 115 articles that failed to meet eligibility criteria: 32 employed non-RCT designs (including observational studies, case series, quasi-experimental designs, and uncontrolled trials), 54 addressed topics outside our research focus (including non-KOA populations, surgical intervention studies, primarily pharmacological treatment investigations, and intervention without proprioceptive exercise), and seven lacked sufficient outcome data. The final analysis included 22 randomized controlled trials (21, 23, 35–54) (Figure 1).

The included studies spanned from 2008 to 2024, representing nine countries and regions. Geographic distribution analysis revealed predominance from India (7/22), the United States (3/22), and Taiwan, China (3/22) (Table 2). Proprioceptive interventions varied methodologically, encompassing proprioceptive training, dynamic stabilization exercises, sensorimotor training, and neuromuscular training. These approaches typically incorporated balance and coordination components and were administered either as primary interventions or as adjuncts to conventional therapy (Table 2). Intervention duration was predominantly short-term, with 17 of 22 studies (21, 23, 35–37, 41–47, 49, 50, 52–54) implementing protocols of 8 weeks or less, while the most extended intervention lasted 12 months (Table 2).

**Table 2:** Characteristic of included studies.

Author	Year	Country/region	Study groups	Comparator	Intervention	Frequency ofintervention	Duration per session	Intervention duration	Implementation of blinding	Outcomes
Apparao et al.	2017	India	CG (48) vs. EG (45)	conventional PT	stabilization exercises	3 sessions per week	not reported	8 weeks	Partial Blinding	VAS
Arif et al.	2022	Pakistan	CG (42) vs. EG (42)	conventional PT	PrT	3 sessions per week	30 min	6 weeks	Non-Blinded	WOMAC-pain WOMAC-function NPRS
Bhaskar et al.	2019	India	CG (15) vs. EG (15)	conventional PT	conventional PT + perturbation training	every other day	not reported	4 weeks	Non-Blinded	TUG
Duman et al.	2012	Turkey	CG (24) vs. EG (30)	conventional PT	PrT	5 sessions per week	not reported	3 weeks	Non-Blinded	WOMAC-total WOMAC-pain WOMAC-stiffness WOMAC-function
Fitzgerald et al.	2011	USA	CG (84) vs. EG (75)	conventional PT	conventional PT + KBA + perturbation techniques	a total of 12 sessions	10–15 min	6–8 weeks	Single-Blind	WOMAC-total WOMAC-function
Gomiero et al.	2018	Brazil	CG (32) vs. EG (32)	RT	SMT	twice a week	not reported	16 weeks	Single-Blind	WOMAC-total VAS TUG
Hale et al.	2012	USA	CG (15) vs. EG (20)	SeniorNet/a card-making activity/Mahjong	water-based PrT	twice a week	20–60 min	12 weeks	Non-Blinded	WOMAC-pain WOMAC-function TUG
Hussein et al.	2015	Egypt	CG (21) vs. EG (38)	conventional PT + acetaminophens	conventional PT + balance training	3 sessions per week	10–15 min	8 weeks	Non-Blinded	VAS
Jahanjoo et al.	2019	Iran	CG (30) vs. EG (30)	conventional PT	conventional PT + balance training	twice a week	60 min	5 weeks	Non-Blinded	WOMAC-total WOMAC-pain WOMAC-stiffness WOMAC-function VAS TUG
Joshi & Kolke	2023	India	CG (26) vs. EG (28)	conventional PT + ST	conventional PT + NMT	twice a week	40 min	6 weeks	Single-Blind	WOMAC-total NRS TUG
Kumar et al.	2013	India	CG (22) vs. EG (22)	conventional PT	conventional PT + PrT	3 sessions per week	not reported	4 weeks	Partial Blinding	NRS
Kuş et al.	2023	Turkey	CG (24) vs. EG (24)	RT	SMT	3 sessions per week	45 min	8 weeks	Non-Blinded	WOMAC-total
Lin et al.	2009	Taiwan, China	CG (36) vs. EG1 (36) vs. EG2 (36)	standard care	EG1: PrT EG2: ST	3 sessions per week	40 min	8 weeks	Non-Blinded	WOMAC-pain WOMAC-function
Oh et al.	2020	Korea	CG (13) vs. EG (13)	conventional PT + ST	conventional PT + visual feedback training	3 sessions per week	not reported	8 weeks	Non-Blinded	VAS
Ojoawo et al.	2016	Nigeria	CG (22) vs. EG (23)	conventional PT + ST	conventional PT + PrT	twice a week	not reported	6 weeks	Non-Blinded	WOMAC-pain WOMAC-stiffness WOMAC-function
Rashid et al.	2019	India	CG (28) vs. EG (31)	QT	NMT	3 sessions per week	30–40 min	12 weeks	Non-Blinded	WOMAC-total
Rathwa et al.	2019	India	CG (30) vs. EG (30)	open kinematic chain exercises	PrT	1 set twice a day for the 1st week; 2 sets twice a day until the 3rd week; 3 sets twice a day until the 5th weeks	not reported	5 weeks	Partial Blinding	WOMAC-total NRS
Rogers et al.	2012	USA	CG (8) vs. EG1 (8) vs. EG2 (8) vs. EG3 (9)	topical cream	EG1: KBA EG2: RT EG3: KBA + RT	3 sessions per week	not reported	8 weeks	Single-Blind	WOMAC-total WOMAC-pain WOMAC-stiffness WOMAC-function
Sharma et al.	2018	India	CG (20) vs. EG (31)	conventional PT + ST	conventional PT + KBA	26 sessions for 12months (baseline visit, 3rd, 7th, 14th, and 21st day from baseline visit); once a week for next 2 months; once every 2 weeks for next 4 months; once a month for next 4 months	not reported	12 months	Partial Blinding	WOMAC-total VAS TUG
Sobhani et al.	2024	Iran	CG (27) vs. EG (27)	conventional PT + ST	conventional PT + PrT	a total of 15 sessions	not reported	6 weeks	Double-blind	VAS
Tsauo et al.	2008	Taiwan, China	CG (14) vs. EG (15)	conventional PT	conventional PT + SMT	3 sessions per week	not reported	8 weeks	Partial Blinding	WOMAC-pain WOMAC-stiffness WOMAC-function
Tudpor et al.	2021	Thailand	CG (7) vs. EG (7)	conventional PT	conventional PT + SEBTx	3 sessions per week	30 min	4 weeks	Non-Blinded	NRS TUG

CG, control group; EG, experimental group; PT, physical therapy; PrT, proprioceptive training; KBA, kinesthesia, balance and agility exercise; SMT, sensorimotor Training; RT, resistance training; NMT, neuromuscular training; ST, strength training; QT, quadriceps training; NRS, numerical rating scale; VAS, visual analog scale; WOMAC, Western Ontario and McMaster universities osteoarthritis index; TUG, timed up and go; SEBTx, star excursion balance test exercise.

### 3.2 Risk of bias

**Figure 2:**
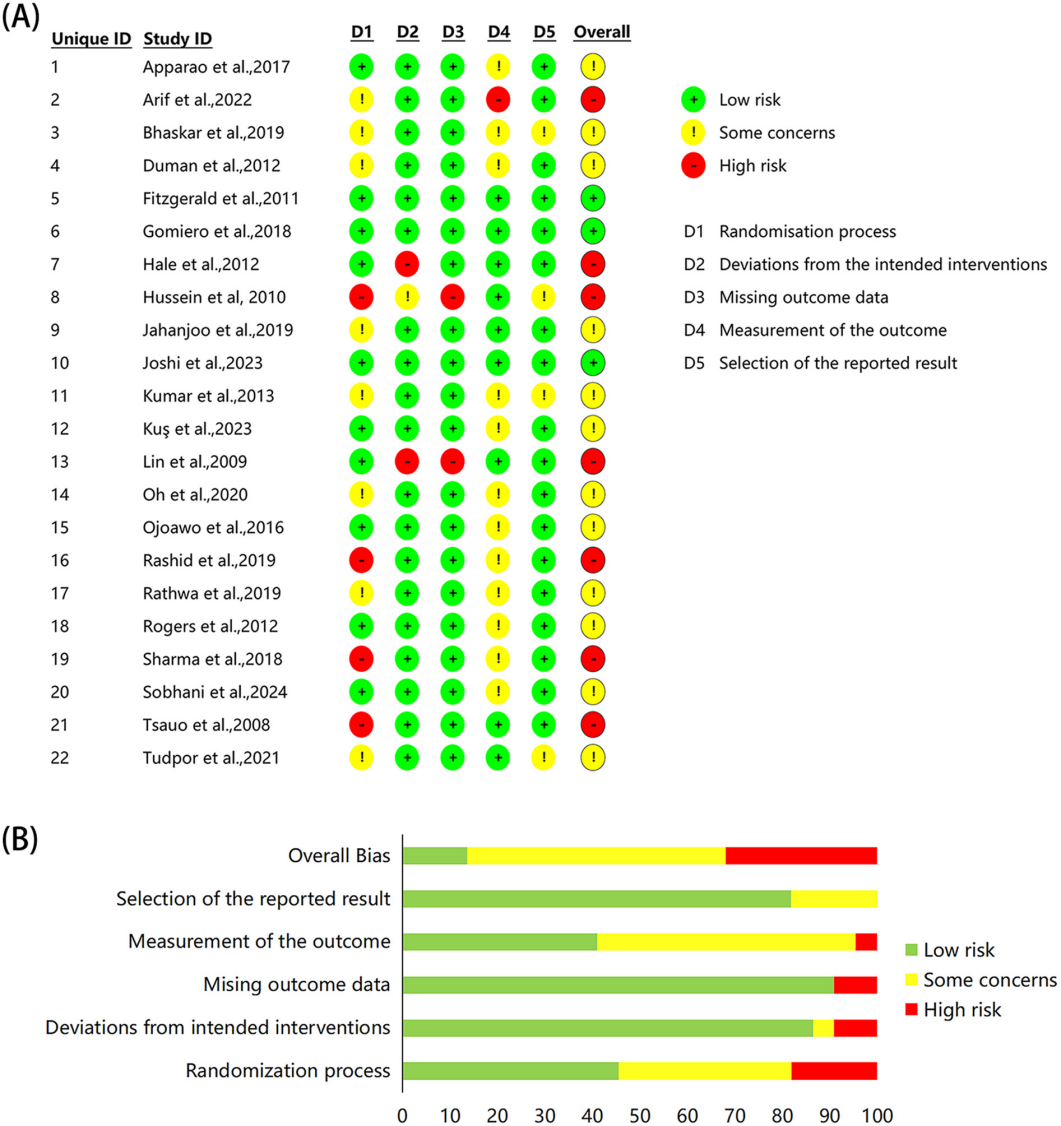
Risk of bias assessment of the included studies. **(A)** Individual study assessment, **(B)** overall proportions across domains.

The 22 studies assessed for risk of bias revealed variable levels of quality across different domains (Figure 2). Ten studies demonstrated low risk of bias in the randomization process, eight studies exhibited some concern, and four studies were classified as high risk due to inadequate randomization details. In terms of deviation from the intended intervention, 19 studies were deemed low risk, while one study exhibited some concerns and two studies were considered high risk. With regard to the completeness of outcome data, 20 studies were classified as low risk, while only two studies were deemed high risk due to incomplete follow-up data. In terms of outcome measures, nine studies were rated as low risk, 12 studies exhibited some concern, and one study was categorized as high risk. Regarding the choice of reporting outcomes, 18 studies were considered low risk, while four studies were assigned some concern due to the absence of a pre-specified analysis plan. The overall assessment indicated that the majority of studies (21, 23, 35, 37, 42, 44, 45, 47, 49, 50, 52, 54) exhibited some concern regarding risk of bias, while seven studies (36, 40, 41, 46, 48, 51, 53) were categorized as high risk and only three studies (38, 39, 43) achieved low risk status.

### 3.3 TUG

**Figure 3:**
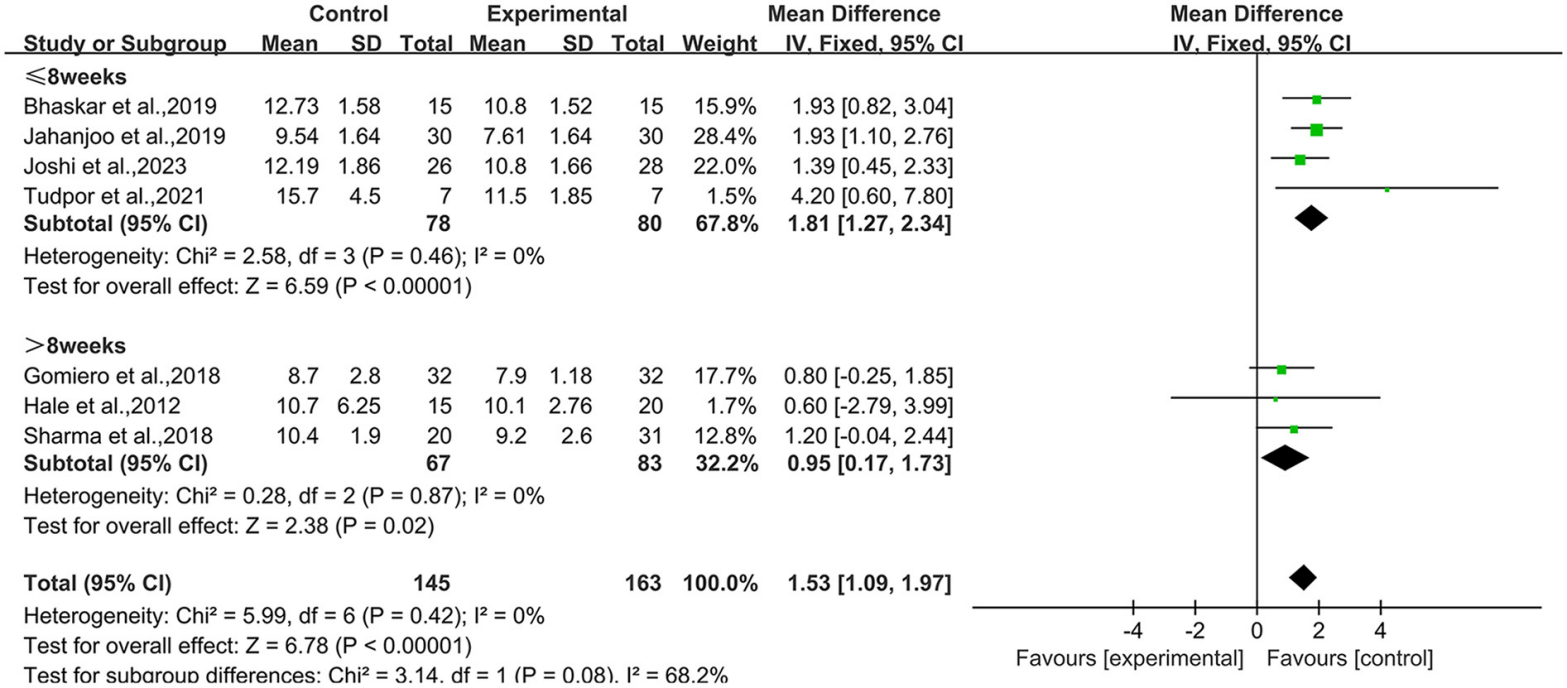
Forest plot of TUG comparing experimental group with control group.

Seven studies (37, 39, 40, 42, 43, 51, 54) reported TUG results in the outcome metrics, involving 308 participants. The experimental group demonstrated a significant improvement in performance on the TUG test compared with the control group [MD = 1.53, 95% CI (1.09, 1.97), *I*^2^ = 0%, *P* < 0.00001, Figure 3]. This improvement exceeded the established minimal clinically important difference (MCID) threshold of 1.3 s (55). The pre-set subgroup analyses revealed that the group receiving interventions duration of ≤8 weeks exhibited a significant improvement [MD = 1.81, 95% CI (1.27, 2.34), *I*^2^ = 0%, *P* < 0.00001, Figure 3], while group with an intervention duration of >8 weeks also demonstrated a statistically significant improvement [MD = 0.95, 95% CI (0.17, 1.73), *I*^2^ = 0%, *P* = 0.02, Figure 3]. Sensitivity analyses indicated good robustness of the results (Supplementary Figure S1A). When assessing the possibility of publication bias, the Egger's test results showed no significant bias (*P* = 0.740 > 0.05, Supplementary Figure S1B).

### 3.4 WOMAC

#### 3.4.1 WOMAC-total

**Figure 4:**
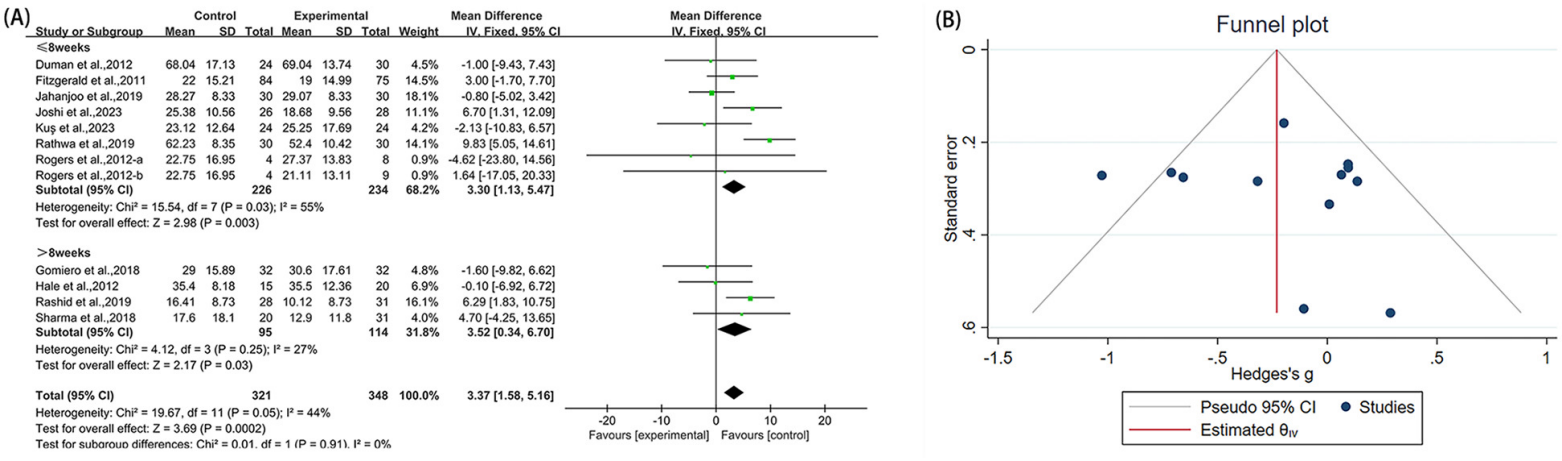
Forest plot and funnel plot of WOMAC-total comparing experimental group with control group, **(A)** forest plot, **(B)** funnel plot.

Twelve studies (23, 38–40, 42, 43, 45, 48–51) reported WOMAC-total in the outcome metrics, involving 669 participants. The experimental group demonstrated a significant reduction in WOMAC-total scores compared with the control group [MD = 3.37, 95% CI (1.58, 5.16), *I*^2^ = 44%, *P* = 0.0002, Figure 4A]. However, this improvement did not reach the established MCID threshold of 4.9 points (56). The pre-set subgroup analyses revealed that the group receiving interventions duration of ≤8 weeks showed a significant improvement [MD = 3.30, 95% CI (1.13, 5.47), *I*^2^ = 55%, *P* = 0.003, Figure 4A], while the group with an intervention duration of >8 weeks also demonstrated a statistically significant improvement [MD = 3.52, 95% CI (0.34, 6.70), *I*^2^ = 27%, *P* = 0.03, Figure 4A]. Sensitivity analyses indicated good robustness of the results (Supplementary Figure S2A). When assessing the possibility of publication bias, both funnel plot analysis (Figure 4B) and Egger's test results showed no significant bias (*P* = 0.252 > 0.05, Supplementary Figure S2B).

#### 3.4.2 WOMAC-pain

**Figure 5:**
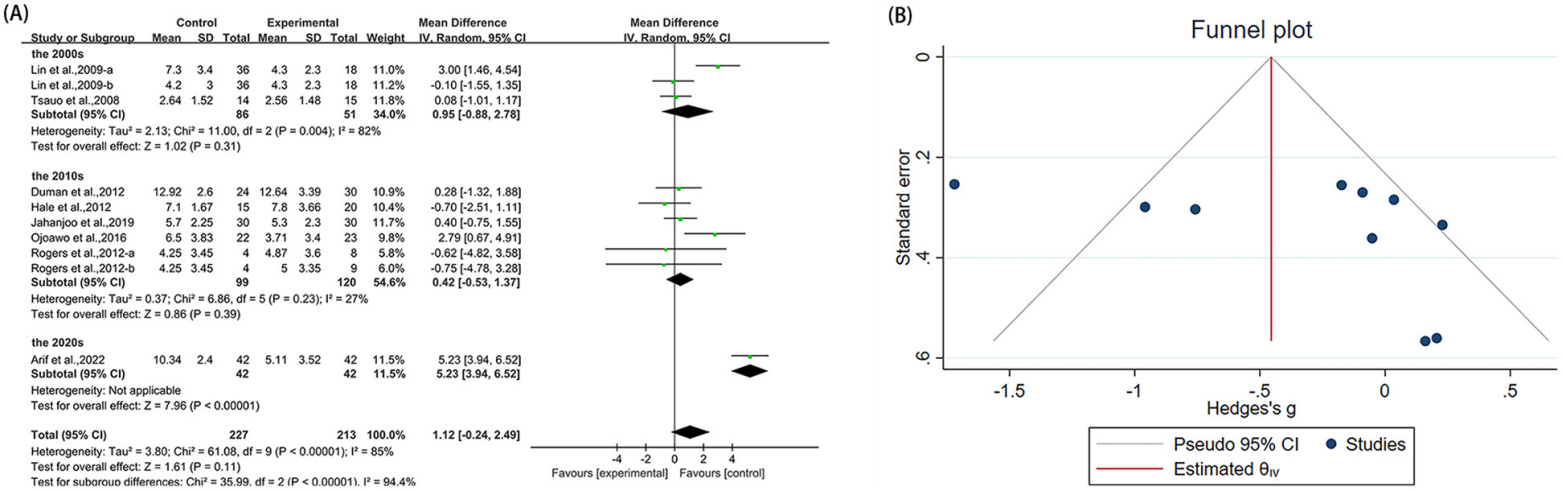
Forest plot and funnel plot of WOMAC-pain comparing experimental group with control group, **(A)** forest plot, **(B)** funnel plot.

Ten studies (21, 23, 36, 40, 42, 46, 50, 53) reported WOMAC-pain in the outcome metrics, involving 440 participants. The experimental group did not demonstrate statistically significant alterations in WOMAC-pain scores compared with the control group [MD = 1.12, 95% CI (−0.24, 2.49), *I*^2^ = 85%, *P* = 0.11, Figure 5A]. We explored sources of high heterogeneity, and subgroup analyses based on publication years found that studies of the 2000s [MD = 0.95, 95% CI (−0.88, 2.78), *I*^2^ = 82%, *P* = 0.31, Figure 5A] and the 2010s showed no significant improvement [MD = 0.42, 95% CI (−0.53, 1.37), *I*^2^ = 27%, *P* = 0.39, Figure 5A]. The 2020s group included only one study that showed significant improvement [MD = 5.23, 95% CI (3.94, 6.52), Figure 5A]. Subgroup analyses based on study region (Asian/non-Asian) and intervention type (single/combined) are presented in the Supplementary Figures S8A, S9A. Sensitivity analyses indicated good robustness of the results (Supplementary Figure S3A). When assessing the possibility of publication bias, both funnel plot analysis (Figure 5B) and Egger's test results showed no significant bias (*P* = 0.785 > 0.05, Supplementary Figure S3B).

#### 3.4.3 WOMAC-stiffness

**Figure 6:**
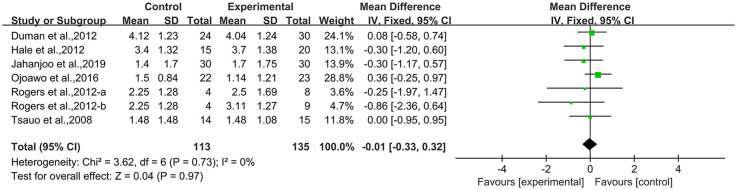
Forest plot of WOMAC-stiffness comparing experimental group with control group.

Seven studies (21, 23, 40, 42, 50, 53) employed WOMAC-stiffness as an outcome measure, involving 248 participants. The experimental group did not demonstrate statistically significant alterations in WOMAC-stiffness scores compared with the control group [MD = −0.01, 95% CI (−0.33, 0.32), *I*^2^ = 0%, *P* = 0.97, Figure 6A]. Sensitivity analyses demonstrated that the overall effect estimate remained stable after sequential exclusion of individual studies, supporting the robustness of the conclusion (Supplementary Figure S4A). Assessment of publication bias revealed potential risks through Egger's test (*P* = 0.041 < 0.05, Supplementary Figure S4B).

#### 3.4.4 WOMAC-function

**Figure 7:**
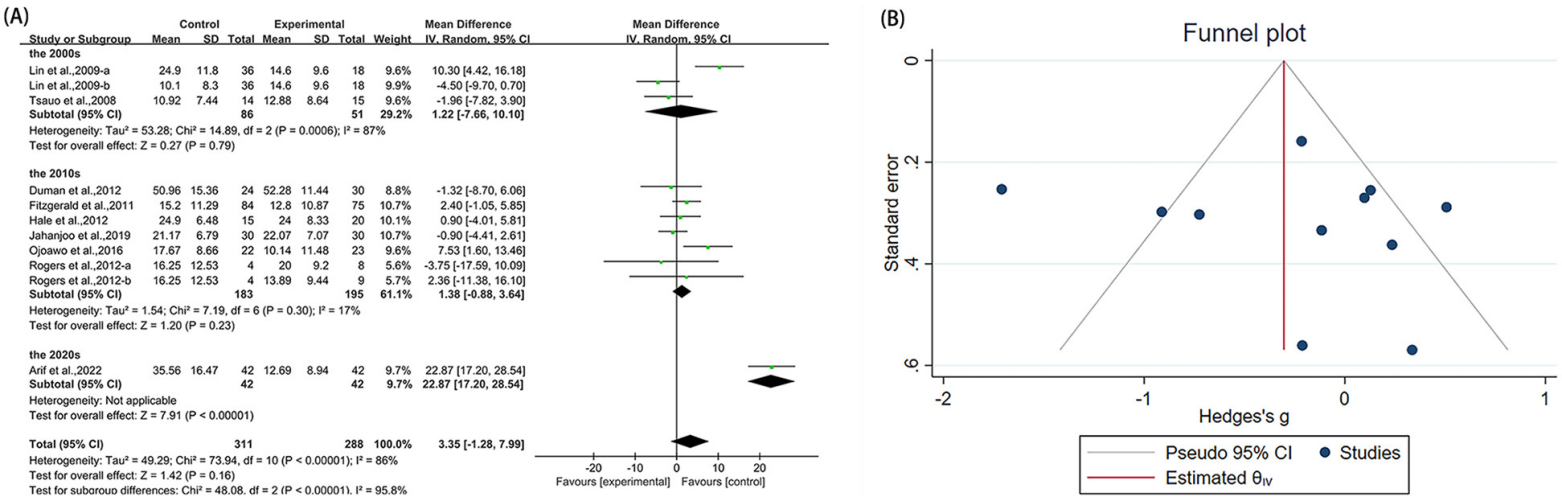
Forest plot and funnel plot of WOMAC-function comparing experimental group with control group, **(A)** forest plot, **(B)** funnel plot.

Eleven studies (21, 23, 36, 38, 40, 42, 46, 50, 53) utilized WOMAC-function as an outcome measure, involving 599 participants. The experimental group did not demonstrate statistically significant alterations in WOMAC-function scores compared with the control group [MD = 3.35, 95% CI (−1.28, 7.99), *I*^2^ = 86%, *P* = 0.16, Figure 7A]. We explored sources of high heterogeneity, and subgroup analyses based on publication years found that studies of the 2000s [MD = 1.22, 95% CI (−7.66, 10.10), *I*^2^ = 87%, *P* = 0.79, Figure 7A] and the 2010s [MD = 1.38, 95% CI (−0.88, 3.64), *I*^2^ = 17%, *P* = 0.23, Figure 7A] showed no significant improvement. The 2020s group included only one study that showed significant improvement [MD = 22.87, 95% CI (17.20, 28.54), Figure 7A]. Subgroup analyses based on study region (Asian/non-Asian) and intervention type (single/combined) are presented in the Supplementary Figures S8B, S9B. Sensitivity analyses confirmed minimal changes to effect estimates upon study exclusion, reinforcing result stability (Supplementary Figure S5A). Both funnel plot symmetry (Figure 7B) and Egger's test (*P* = 0.790 > 0.05, Supplementary Figure S5B) showed no evidence of publication bias.

### 3.5 Pain score

#### 3.5.1 NRS

**Figure 8:**
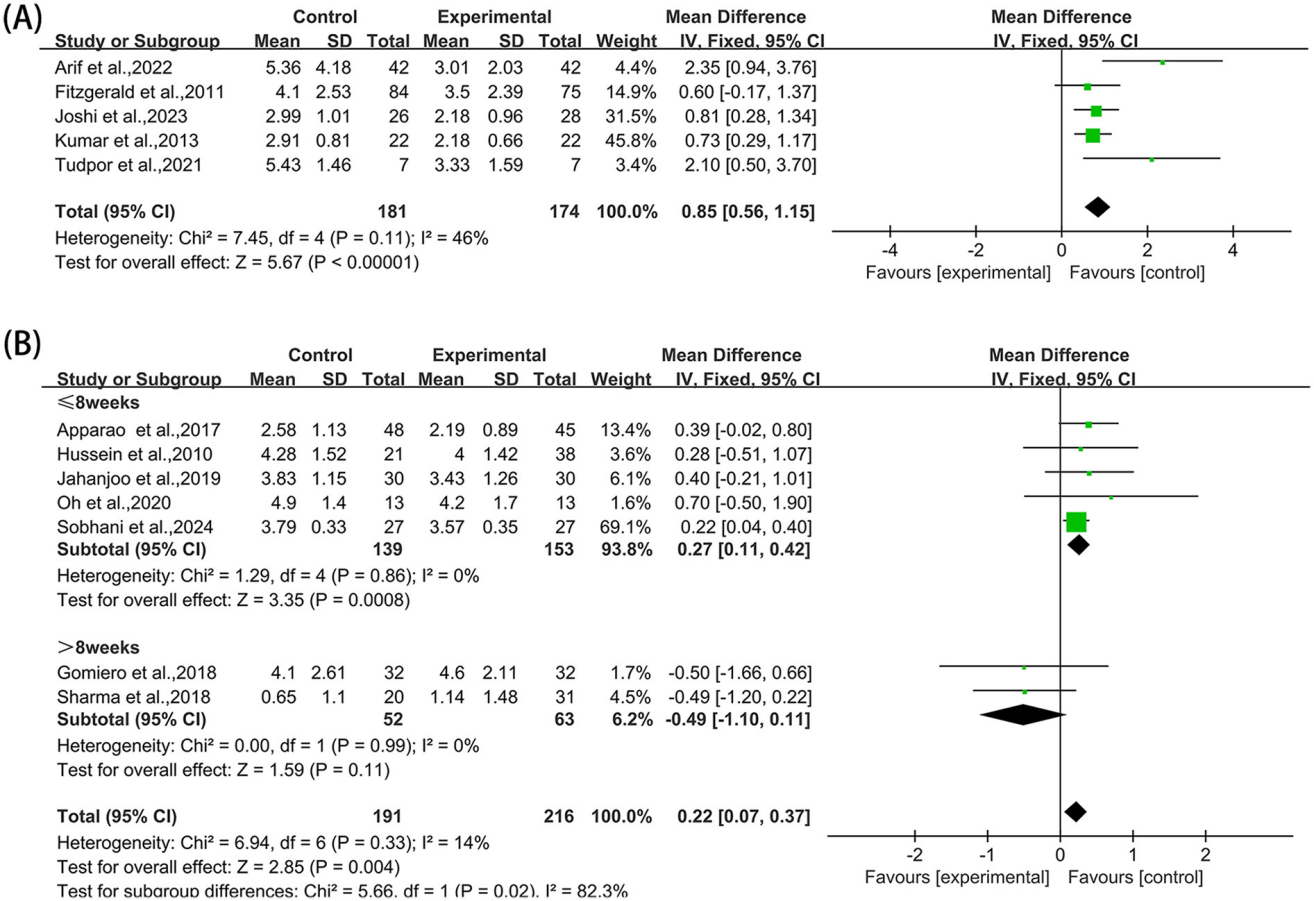
Forest plot of NRS and VAS comparing experimental group with control group, **(A)** NRS, **(B)** VAS.

Five studies (36, 38, 43, 44, 54) reported NRS in the outcome metrics, involving 355 participants. The experimental group demonstrated a significant reduction in NRS scores compared with the control group [MD = 0.85, 95% CI (0.56, 1.15), *I*^2^ = 46%, *P* < 0.00001, Figure 8A]. However, this improvement did not reach the established MCID of 1.65 points for NRS scores (57). Given that all studies implemented interventions with a duration of ≤8 weeks, the pre-specified subgroup analyses by duration were not performed. Sensitivity analyses indicated high robustness of the results (Supplementary Figure S6A). When assessing the possibility of publication bias, Egger's test results showed no significant bias (*P* = 0.066 > 0.05, Supplementary Figure S6B).

#### 3.5.2 VAS

Seven studies (35, 39, 41, 42, 47, 51, 52) reported VAS in the outcome metrics, involving 407 participants. A statistically significant decrease in pain scores was observed in the experimental group compared with the control group [MD = 0.22, 95% CI (0.07, 0.37), *I*^2^ = 14%, *P* = 0.004, Figure 8B]. Nevertheless, this reduction fell substantially below the MCID threshold of 1.80 cm for VAS scores (58). The pre-set subgroup analysis showed that the intervention group receiving intervention duration of ≤ 8 weeks exhibited better improvement [MD = 0.27, 95% CI (0.11, 0.42), *I*^2^ = 0%, *P* = 0.0008, Figure 8B], while the intervention group receiving intervention duration of >8 weeks had no statistically significant improvement [MD = −0.49, 95% CI (−1.10, 0.11), *I*^2^ = 0%, *P* = 0.11, Figure 8B]. Sensitivity analyses substantiated the robustness of these conclusions (Supplementary Figure S7A). No significant publication bias was identified through Egger's test (*P* = 0.671 > 0.05, Supplementary Figure S7B).

### 3.6 Certainty of evidence

The certainty of evidence was assessed using the GRADE approach. Overall, the quality of evidence was rated as low due to concerns regarding risk of bias across multiple domains and potential publication bias detected for WOMAC-stiffness outcomes, resulting in a downgrade of two levels from the initial high quality rating.

## 4 Discussion

This meta-analysis demonstrates that proprioceptive exercise provides beneficial effects for patients with KOA. The most pronounced improvement was observed in balance function as measured by the TUG test, reaching clinically meaningful levels. While statistically significant reductions were observed in overall WOMAC-total scores and pain intensity measures (NRS and VAS), these improvements fell below established minimal clinically important difference thresholds. No significant improvements were found for individual WOMAC subscales (pain, stiffness, or function). Proprioceptive exercise has increasingly become a focus of scholarly investigation in KOA management. Prior systematic reviews and meta-analyses have demonstrated that proprioceptive exercises may enhance joint position sense, reduce pain intensity, and offer some other therapeutic benefits for patients with KOA (24–26). Nevertheless, empirical evidence specifically addressing balance function outcomes has remained insufficiently examined in the literature. In addressing this gap in the literature, our research has sought to contribute to the body of knowledge by including a more comprehensive array of randomized controlled trials. These trials have enabled the investigation of the effects of proprioceptive exercise on pain, overall symptoms, and function, as well as its association with improved balance capabilities.

This study is the first meta-analysis to assess the impact of proprioceptive exercises on balance capabilities in patients with KOA. This clinical parameter has received scant attention in previous research. The TUG test improvements demonstrated both statistical significance and clinical meaningfulness, with effect sizes exceeding the established MCID threshold, indicating that the changes translate into meaningful functional benefits for patients. These improvements translate into increased confidence and efficiency during activities of daily living, including sit-stand transitions, walking, and turning. They also provide increased stability during orientation changes (59, 60). The underlying neural mechanisms extend beyond peripheral improvements to include significant central nervous system adaptations. Research has demonstrated that proprioceptive exercise modifies spinal reflex circuits, as evidenced by changes in H-reflex amplitudes, indicating altered processing of afferent inputs at the spinal level (61). At supraspinal levels, studies utilizing transcranial magnetic stimulation have revealed reduced corticospinal excitability and enhanced intracortical inhibition following proprioceptive training, particularly during postural responses. These cortical adaptations exhibit a strong correlation with improvements in postural stability, suggesting that optimized sensory processing in sensorimotor cortical areas plays a critical role in balance enhancement (62). Collectively, these central adaptations facilitate more efficient integration of proprioceptive information and refinement of motor output, improving the body's ability to maintain balance during functional activities (63). Subgroup analysis based on intervention duration revealed significant TUG improvements for both short-term (≤8 weeks) and long-term (>8 weeks) interventions. However, short-term interventions demonstrated larger effect sizes and stronger statistical significance. These findings imply that proprioceptive exercises hold promise in enhancing balance function in patients with KOA, particularly among those with existing balance impairments and elevated fall risks. Moreover, maintaining exercise within an 8-week period may be associated with superior outcomes.

While balance function showed robust improvements, the effects on other clinical outcomes presented a more complex pattern. With regard to the evaluation of KOA symptoms, the analysis of WOMAC-total results indicates that proprioceptive exercises significantly improved the overall condition of patients with KOA, irrespective of intervention duration (*P* = 0.0002). However, the mean difference (MD = 3.37) did not reach the MCID threshold, suggesting that while the improvement was statistically significant, its clinical relevance may be limited. This finding indicates that proprioceptive exercise shows promise for patients with KOA, but modifications may be needed to achieve clinically meaningful outcomes. This finding is consistent with the results of previous research (25, 26). However, pain-related outcome analyses revealed notable variability across different measurement tools. While WOMAC-pain assessments did not reach statistical significance (*P* = 0.11), VAS (*P* = 0.004) and NRS (*P* < 0.00001) demonstrated statistically significant improvements that fell short of MCID thresholds, suggesting that these tools may have different dimensional focuses and sensitivities (64). These findings partially align with Wang et al.'s study though the clinical meaningfulness of such improvements requires careful interpretation considering MCID thresholds (26). VAS and NRS primarily evaluate overall perceived pain intensity, emphasizing immediate, general pain perception (65), whereas WOMAC-pain, as a multidimensional assessment tool, evaluates pain experiences across several daily activities (66). This distinction aligns closely with the characteristic symptom pattern in patients with KOA, where pain typically worsens during activity and diminishes during rest (67, 68). The observed pattern suggests that proprioceptive exercise may have modest effects on general pain perception while potentially having limited impact on activity-specific pain experiences. These findings suggest that multiple pain assessment tools should be considered when evaluating intervention efficacy, with recognition that proprioceptive exercise may primarily benefit balance and functional outcomes rather than serving as a primary pain management strategy.

Subgroup analysis of VAS scores revealed statistical significance for short-term interventions (*P* = 0.0008), but not for long-term interventions (*P* = 0.11). This phenomenon may be attributed to several factors. These include rapid neural adaptations that occur during the early stages of proprioceptive exercise (69). Typically, adherence and motivation of patients exhibit higher levels during initial intervention periods but may gradually diminish with extended intervention duration, potentially influencing the observed outcomes in longer interventions. This phenomenon was potentially exemplified in the study by Sharma et al., which reported relatively high dropout rates, possibly attributable to their extended intervention duration of 12 months (51). Additionally, potential plateau effects where initial benefits stabilize after reaching maximum potential (70). Despite this finding in VAS measurements, it is important to emphasize that other outcome measures, including WOMAC-total and TUG, continued to show benefits beyond 8 weeks, underscoring the efficacy of proprioceptive exercises as a long-term intervention strategy.

The findings indicate a lack of statistical significance in WOMAC-stiffness (*P* = 0.97) and WOMAC-function (*P* = 0.16) analyses, contrasting with the significant improvement observed in WOMAC-total. This phenomenon suggests that proprioceptive exercises may produce broad but modest cumulative effects on the overall condition of patients with KOA. While improvements in individual symptom dimensions may not reach significant thresholds, composite scores appear more sensitive in capturing these multidimensional, incremental improvements.

We employed comprehensive analytical approaches to investigate the sources of heterogeneity. Subgroup analyses based on intervention duration and methodology (single vs. combined interventions) did not adequately explain the high heterogeneity observed in WOMAC-pain results. However, regional subgroup analysis (Asian vs. non-Asian) for WOMAC-function revealed low heterogeneity (*I*^2^ = 0%) and statistical significance (*P* = 0.03) in the non-Asian group, suggesting that geographic factors may significantly influence intervention consistency. The analysis of other potential classification methods, including patient demographics (age, gender, BMI), K-L grading, and specific proprioceptive exercise protocols, was hindered by inconsistent reporting and a lack of standardized information across studies. Future research should standardize reporting of patient demographics, disease severity, and intervention parameters to enable more targeted analyses of which patient subgroups benefit most from proprioceptive exercises.

After extensive exploration, we employed publication era-based subgroup analysis, revealing a meaningful temporal trend. Studies of the 2000s generally exhibited high heterogeneity, with WOMAC-pain (*I*^2^ = 82%) and WOMAC-function (*I*^2^ = 86%) demonstrating significant inter-study differences. Conversely, studies of the 2010s showed improved homogeneity, with heterogeneity substantially reduced to 27% for WOMAC-pain and 17% for WOMAC-function. This heterogeneity trend may signify the field's advancement towards a more consolidated understanding of proprioceptive exercise applications in KOA management, with intervention designs progressively centered on specific mechanisms, thereby yielding more consistent therapeutic effects.

This study is subject to several limitations. Methodologically, the 22 randomized controlled trials included in this analysis demonstrated moderate overall quality, with only thre studies achieving a low risk of bias classification. There was limited implementation of robust blinding procedures across studies, with only one study utilizing a double-blind design, which may introduce potential detection and performance bias, particularly for subjective outcomes such as pain assessment. This limitation could potentially inflate effect sizes for patient-reported measures like VAS and NRS scores, warranting cautious interpretation of these findings. Four studies showed weaknesses in the randomization process due to inadequate reporting of sequence generation or allocation concealment. The heterogeneity of intervention protocols presents another important consideration, as the included studies varied in training content, duration, and methodology. Notably, none of the included studies provided detailed reporting of exercise intensity parameters such as percent of target heart rate or metabolic equivalent (MET) values, which represents a significant gap in standardization of interventions. This variability makes it challenging to formulate specific recommendations for standardized proprioceptive exercise protocols in clinical settings. The inclusion of both no-treatment and active therapy controls in our analysis may have contributed to this pattern of statistical significance without clinical meaningfulness across multiple measures. Additionally, the inconsistent reporting of patient characteristics across studies limits our ability to determine which patient subgroups might benefit most from these interventions. Furthermore, the study's inclusion of English-language publications may introduce a language bias, as it overlooks relevant research published in other languages. While Egger's tests for most outcome measures showed no significant bias, the results for WOMAC-stiffness (*P* = 0.041 < 0.05) indicated potential publication bias. These limitations underscore the necessity for future research to develop standardized proprioceptive exercise protocols through high-quality longitudinal studies. The investigation of individualized intervention approaches, tailored to patient characteristics and disease staging, would be a valuable avenue for future research.

Collectively, these findings indicate that the incorporation of proprioceptive exercises into the rehabilitation of KOA is a crucial component, particularly for patients experiencing knee pain and balance dysfunction. Evidence suggests that the implementation of proprioceptive exercises within an 8-week timeframe may yield optimal therapeutic effects, especially for improving balance function and alleviating pain. Clinical practice should incorporate comprehensive assessment approaches that combine subjective and objective measurements to track therapeutic progress and optimize patient outcomes. These insights provide more comprehensive evidence-based guidance for clinical KOA management practices.

## 5 Conclusion

Based on low-certainty evidence, proprioceptive exercises may improve balance function and overall clinical status in patients with KOA, though further high-quality research is needed to strengthen confidence in these findings.The analysis revealed that short-term interventions yield particularly significant effects. However, none of the WOMAC subscales reached statistical significance individually. Based on these findings, we recommend incorporating proprioceptive exercises into clinical rehabilitation protocols for patients with KOA, especially for those suffering from balance dysfunction.
