# Supplementary figures and images for: Effects of proprioceptive exercise for knee osteoarthritis: a systematic review and meta-analysis
Source: Front Rehabil Sci. 2025 Jun 24;6:1596966. doi: 10.3389/fresc.2025.1596966 (PMC12234485; doi:10.3389/fresc.2025.1596966)

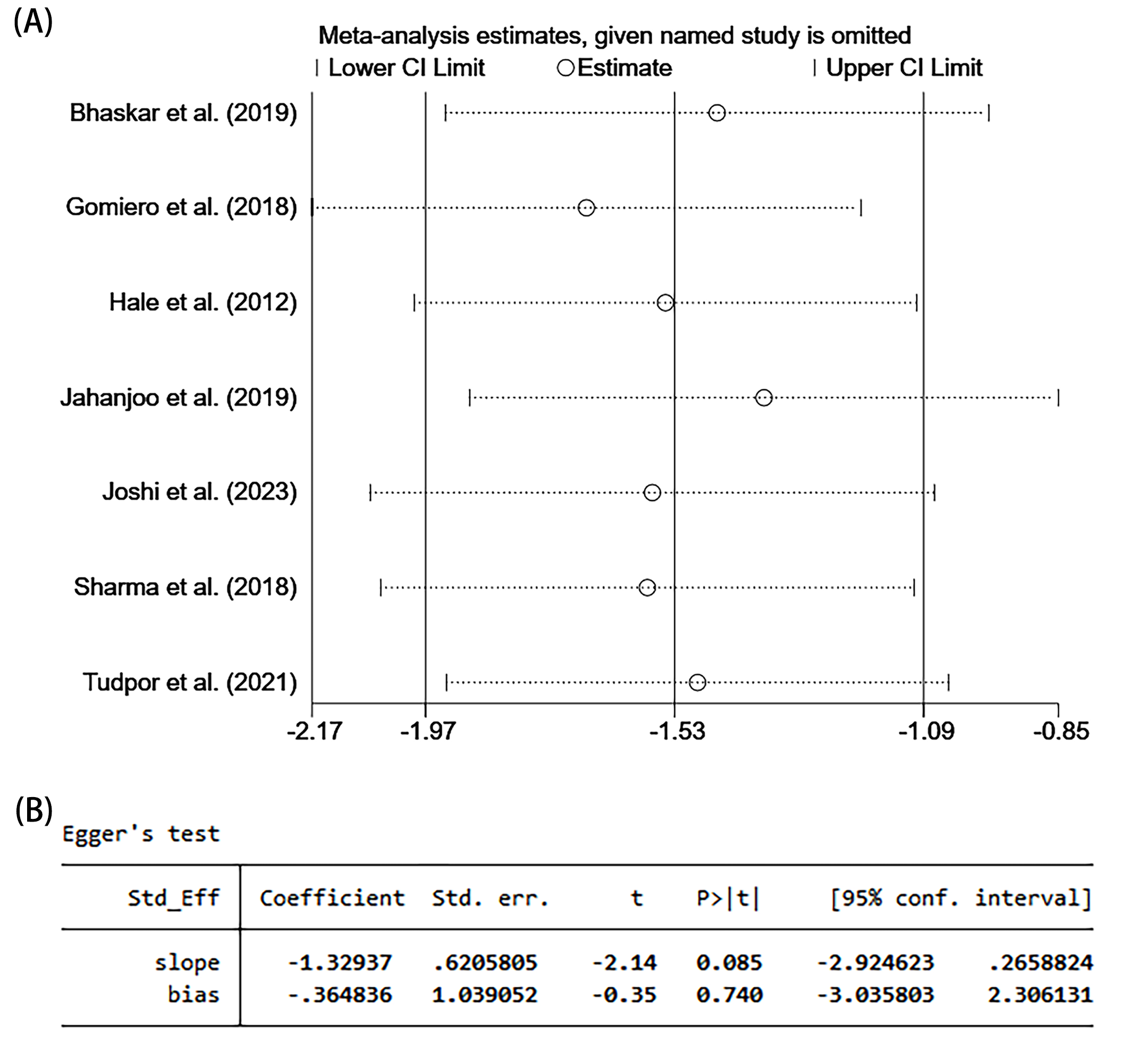

Supplement: Supplementary file 1 [file Image1.tif]

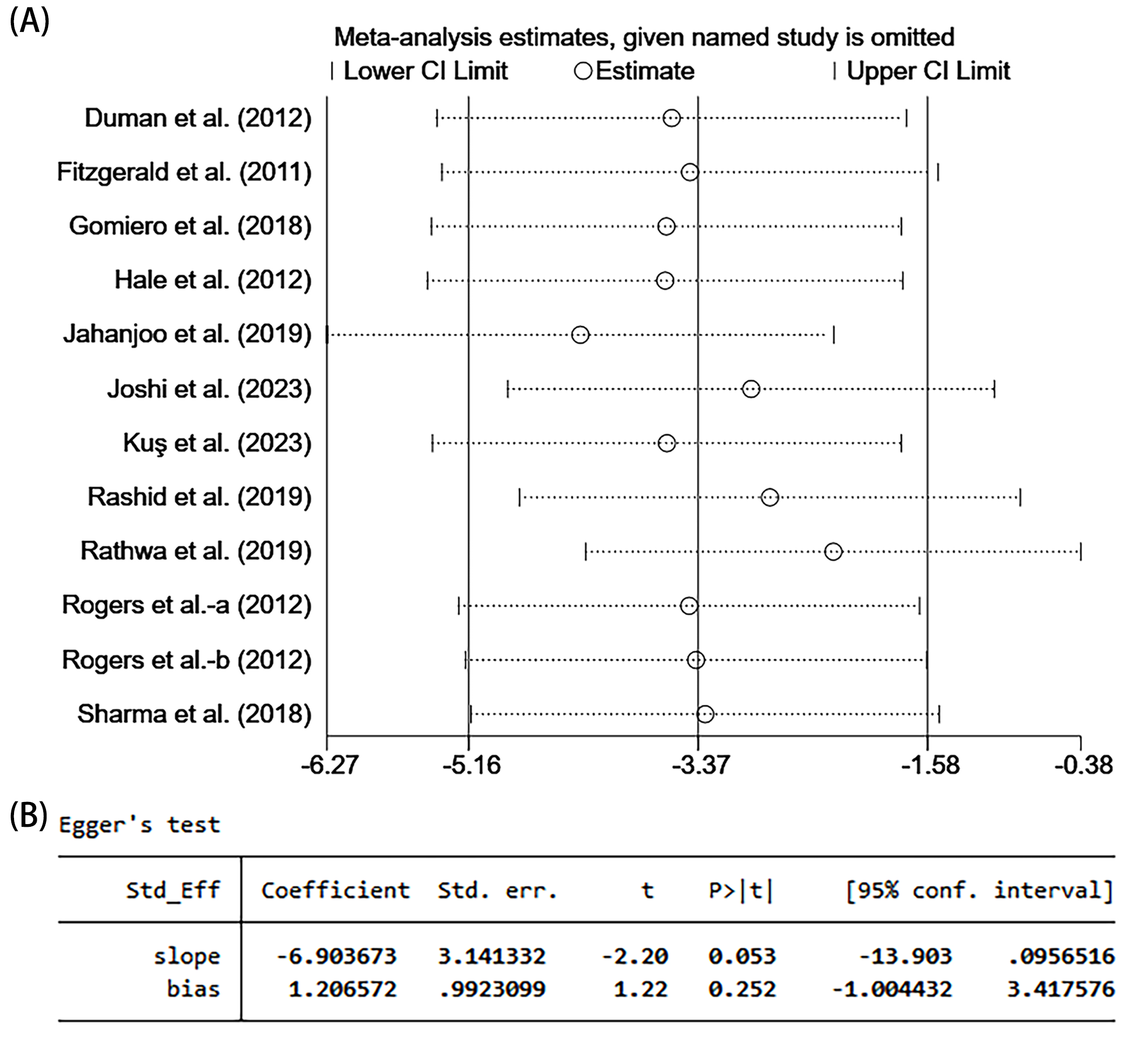

Supplement: Supplementary file 2 [file Image2.tif]

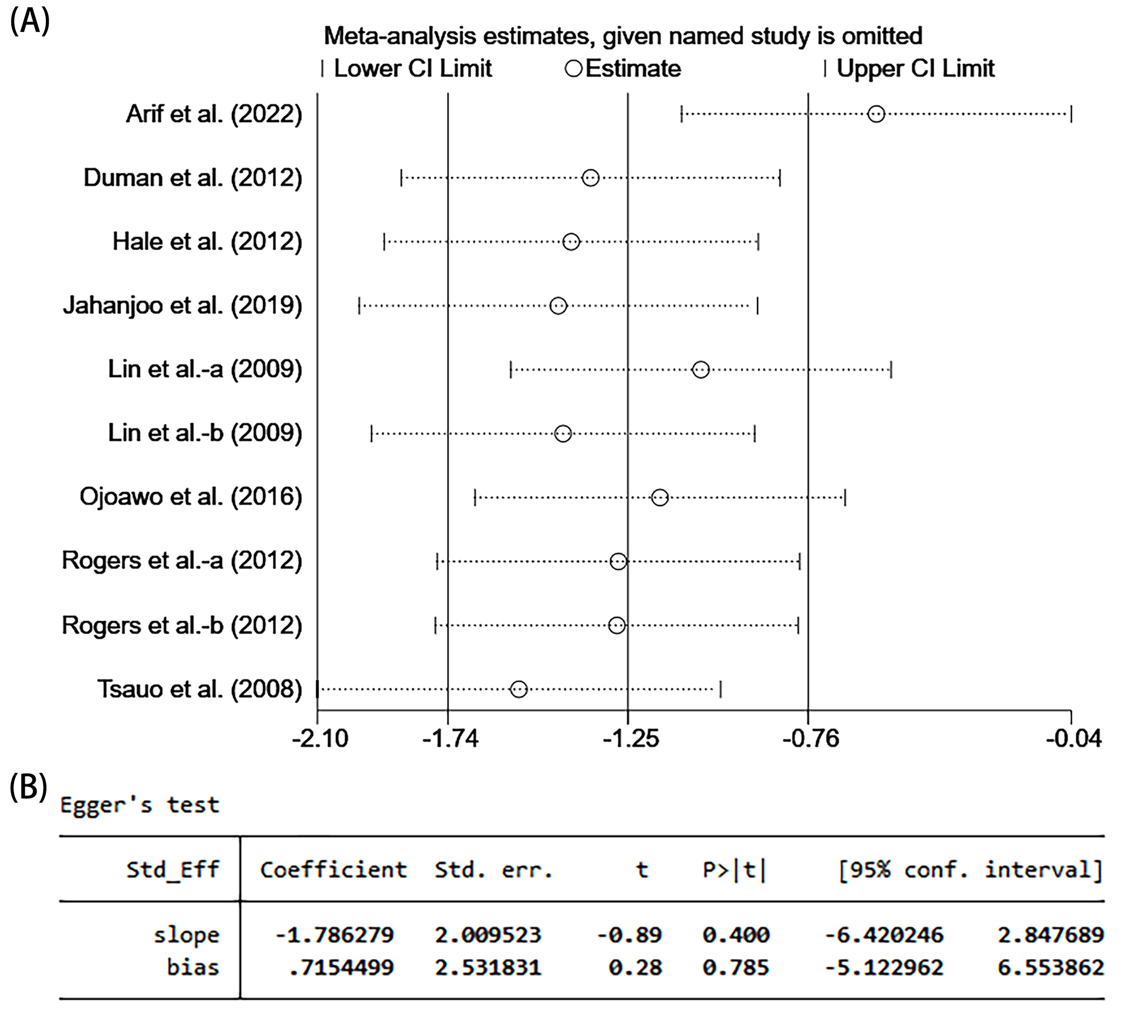

Supplement: Supplementary file 3 [file Image3.tif]

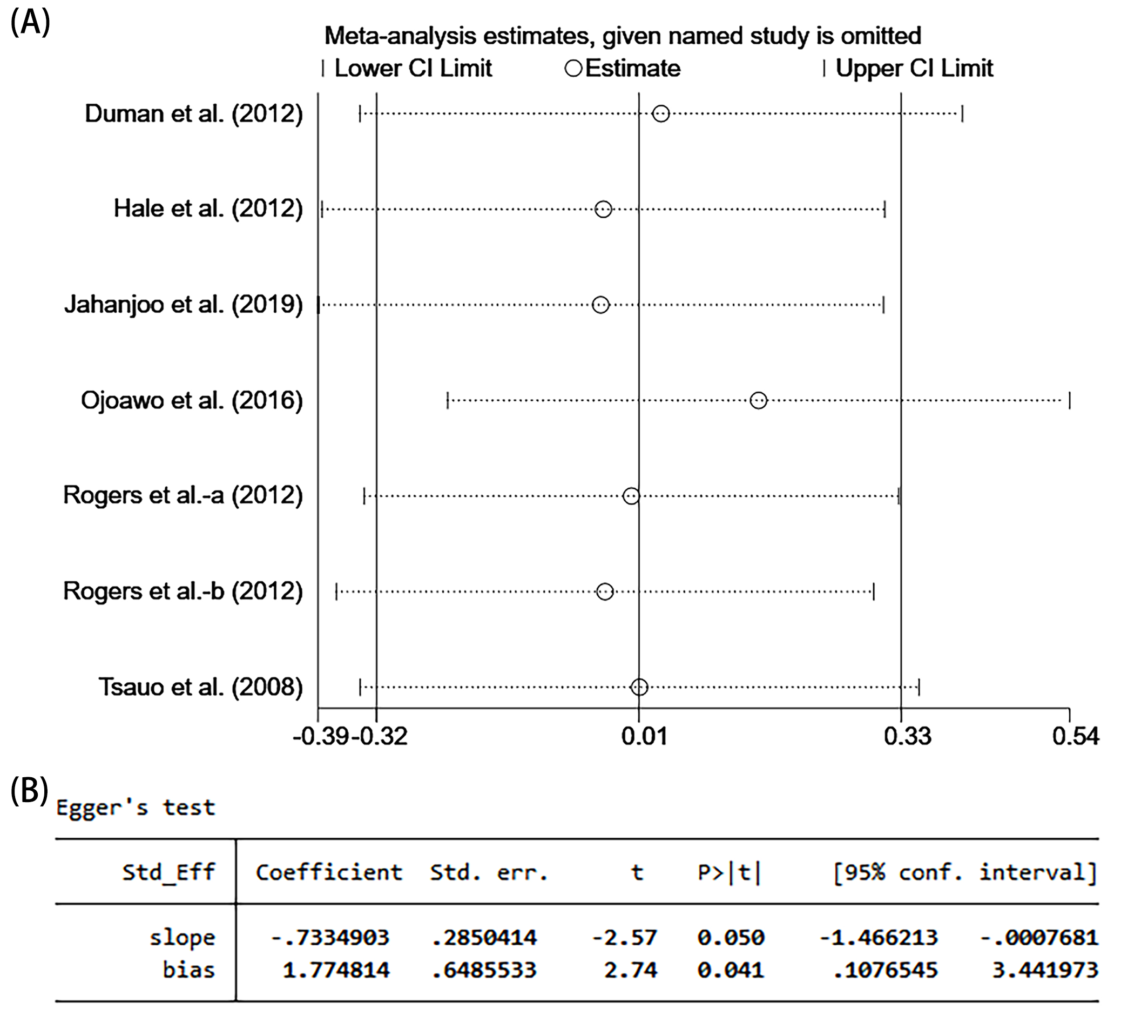

Supplement: Supplementary file 4 [file Image4.tif]

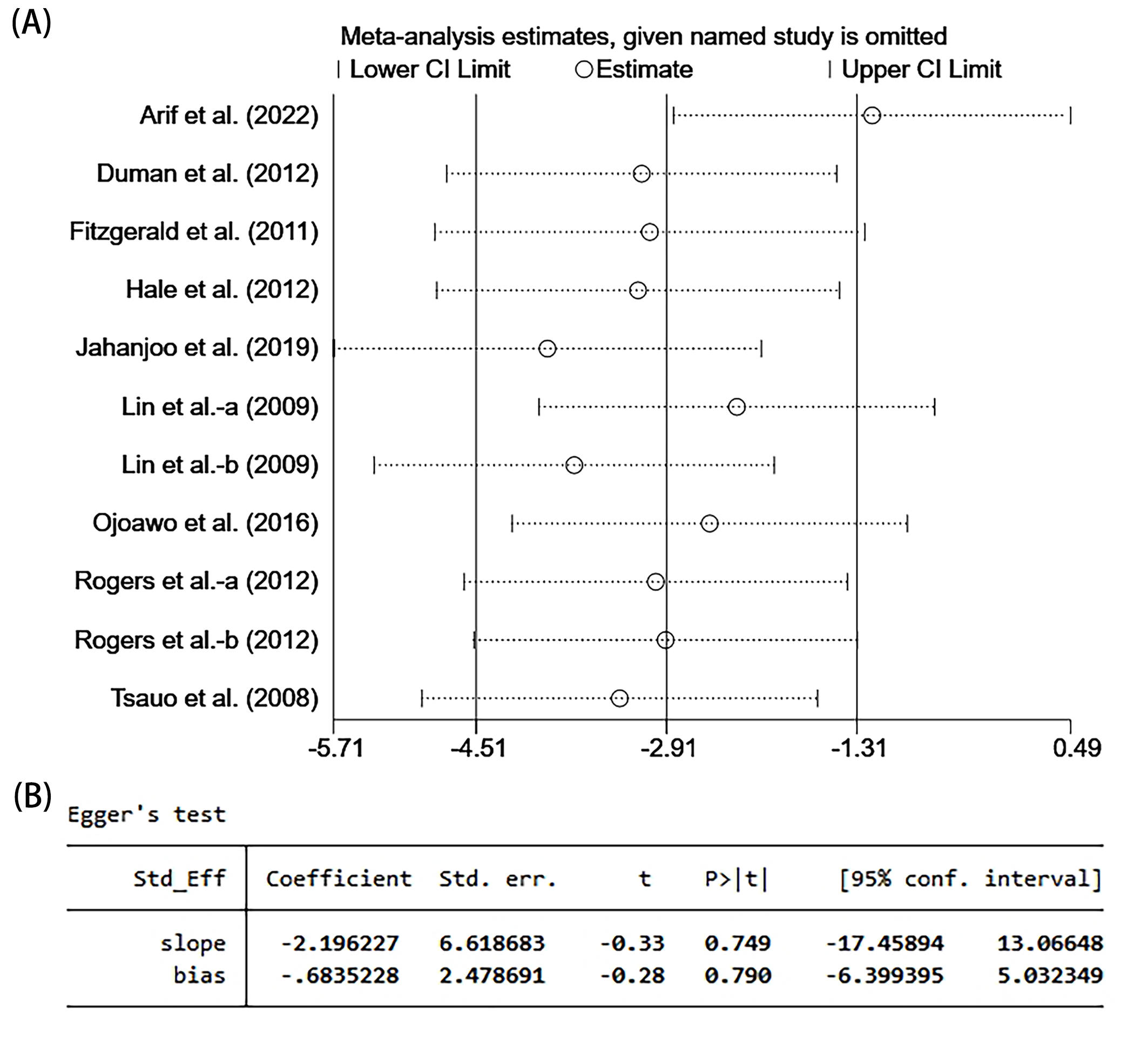

Supplement: Supplementary file 5 [file Image5.tif]

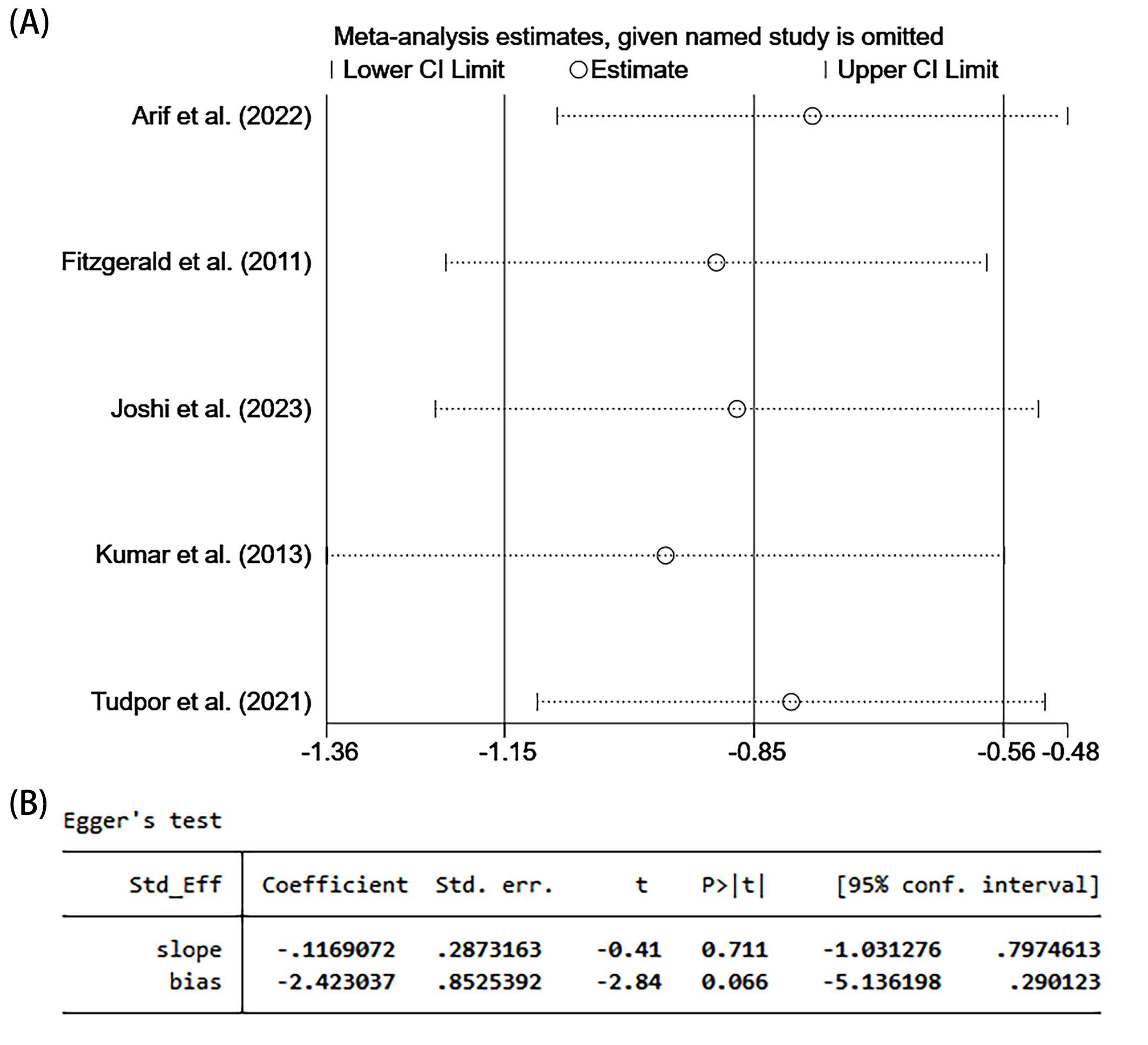

Supplement: Supplementary file 6 [file Image6.tif]

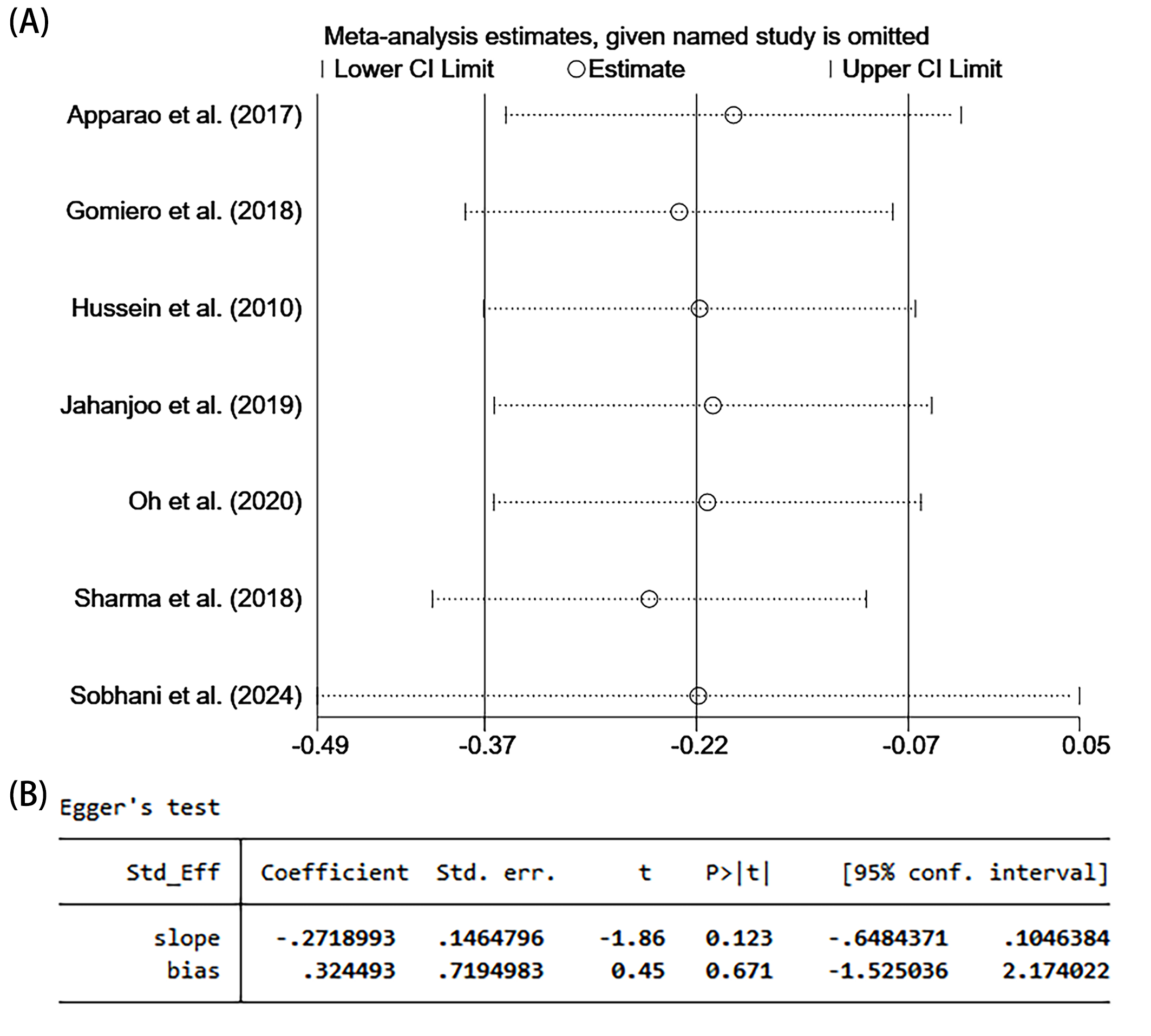

Supplement: Supplementary file 7 [file Image7.tif]

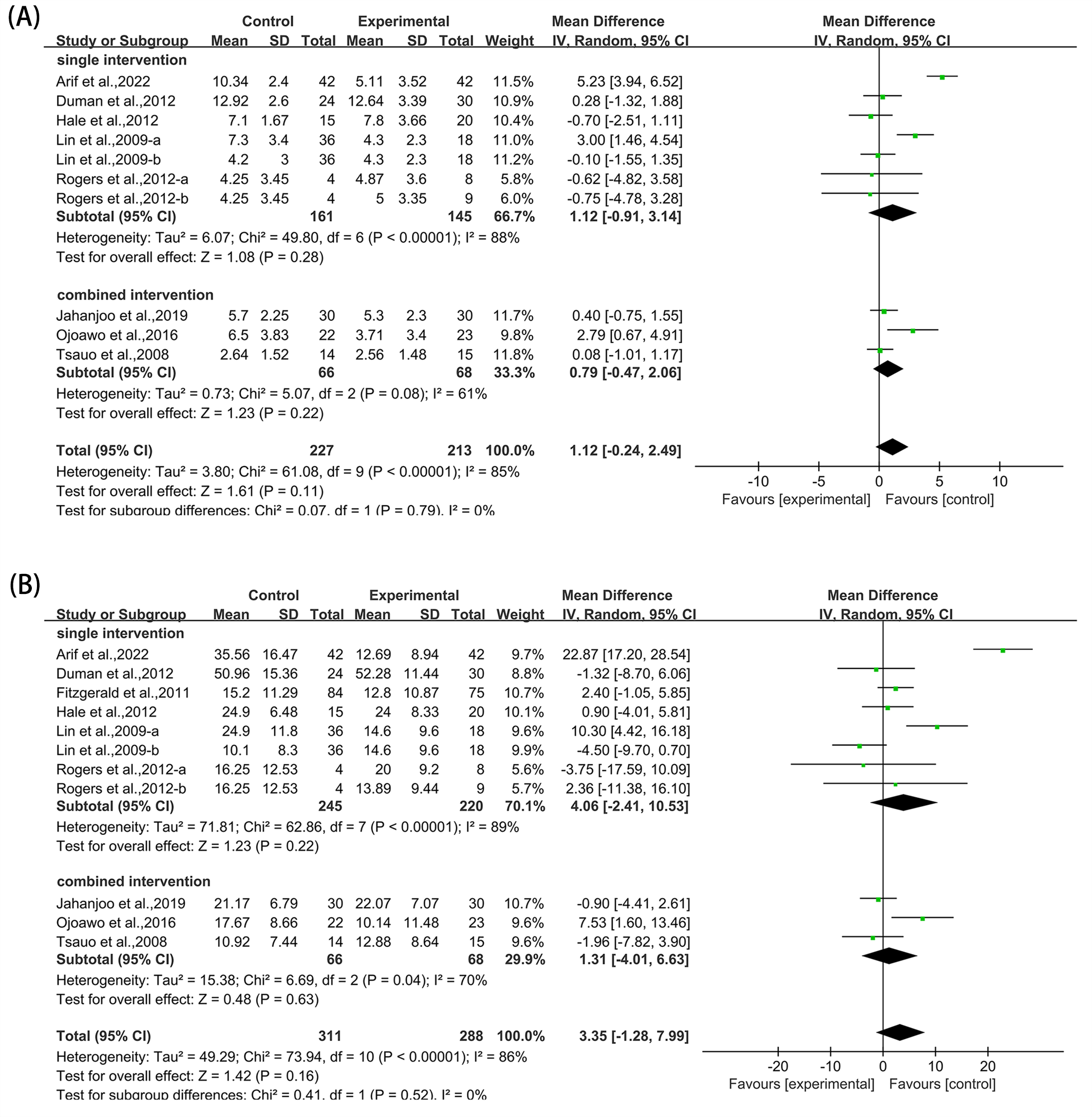

Supplement: Supplementary file 8 [file Image8.tif]

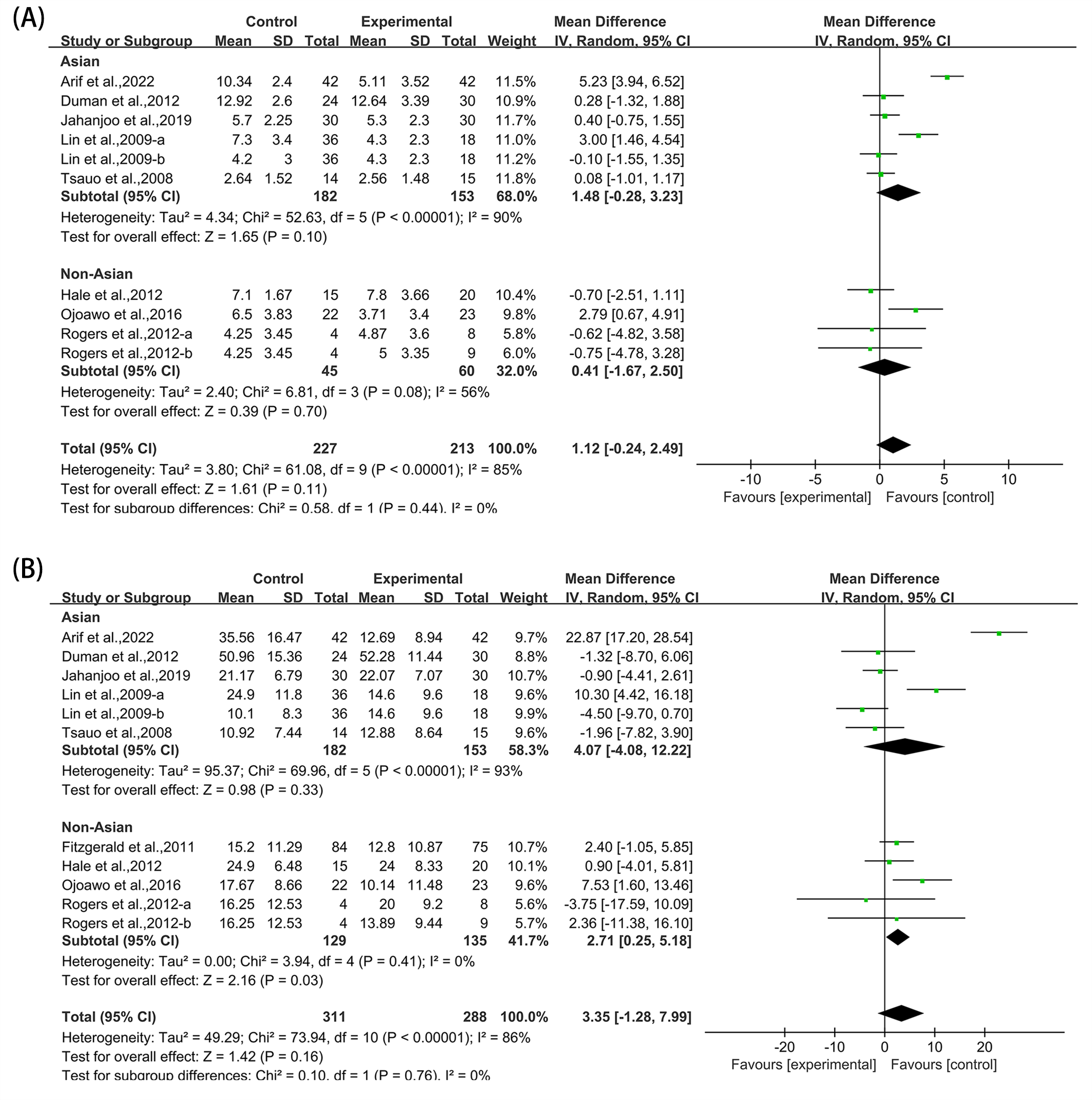

Supplement: Supplementary file 9 [file Image9.tif]
