# Supplementary material for: Effects of proprioceptive exercise for knee osteoarthritis: a systematic review and meta-analysis
Source: Front Rehabil Sci. 2025 Jun 24;6:1596966. doi: 10.3389/fresc.2025.1596966 (PMC12234485; doi:10.3389/fresc.2025.1596966)
Supplement: Supplementary file 10 [file Table1.docx]

**S1: Search Strategy**

**PubMed N=434**

| PubMed Search History | | |
| --- | --- | --- |
| **Search number** | **Query** | **Results** |
| **#1** | "osteoarthritis, knee"[MeSH Terms] | 30327 |
| **#2** | "Osteoarthritis"[MeSH Terms] AND "Knee"[All Fields] | 39770 |
| **#3** | "osteoarthrit*"[Title/Abstract] AND "knee*"[All Fields] | 46703 |
| **#4** | "knee osteoarthritis"[Title/Abstract] | 19157 |
| **#5** | "Osteoarthritis of Knee"[Title/Abstract] | 212 |
| **#6** | "Osteoarthritis of Knees"[Title/Abstract] | 19 |
| **#7** | "Knee OA"[Title/Abstract] | 8516 |
| **#8** | "gonarthrosis"[Title/Abstract] | 1223 |
| **#9** | #1 OR #2 OR #3 OR #4 OR #5 OR #6 OR #7 OR #8 | 56378 |
| **#10** | "Proprioception"[MeSH Terms] | 40564 |
| **#11** | "Kinesthesis"[MeSH Terms] | 3352 |
| **#12** | "Postural Balance"[MeSH Terms] | 30155 |
| **#13** | "propriocep*"[Title/Abstract] | 15755 |
| **#14** | "kinesthes*"[Title/Abstract] | 632 |
| **#15** | "position sense"[Title/Abstract] | 1697 |
| **#16** | "movement sense"[Title/Abstract] | 61 |
| **#17** | "velocity sense"[Title/Abstract] | 5 |
| **#18** | "force sense"[Title/Abstract] | 85 |
| **#19** | "sensorimotor training"[Title/Abstract] | 294 |
| **#20** | "balance training"[Title/Abstract] | 2180 |
| **#21** | "postural control"[Title/Abstract] | 8785 |
| **#22** | "neuromuscular training"[Title/Abstract] | 665 |
| **#23** | "stability training"[Title/Abstract] | 211 |
| **#24** | "kinesthetic training"[Title/Abstract] | 10 |
| **#25** | "core stability"[Title/Abstract] | 816 |
| **#26** | #10 OR #11 OR #12 OR #13 OR #14 OR #15 OR #16 OR #17 OR #18 OR #19 OR #20 OR #21 OR #22 OR #23 OR #24 OR #25 | 55466 |
| **#27** | "Randomized Controlled Trial"[Publication Type] | 631193 |
| **#28** | "Controlled Clinical Trial"[Publication Type] | 721953 |
| **#29** | "randomized"[Title/Abstract] | 740409 |
| **#30** | "randomised"[Title/Abstract] | 144860 |
| **#31** | "randomly"[Title/Abstract] | 451701 |
| **#32** | "trial"[Title/Abstract] | 861490 |
| **#33** | "groups"[Title/Abstract] | 2833232 |
| **#34** | #27 OR #28 OR #29 OR #30 OR #31 OR #32 OR #33 | 4094019 |
| **#35** | #9 AND #26 AND #34 | 434 |

**Scopus N=775**

| Scopus Search History | | |
| --- | --- | --- |
| **Search number** | **Query** | **Results** |
| **#1** | TITLE-ABS-KEY ( "osteoarthritis, knee" ) | 28493 |
| **#2** | ( TITLE-ABS-KEY ( "Osteoarthritis" ) AND ALL ( "Knee" ) ) | 112217 |
| **#3** | ( TITLE-ABS-KEY ( "osteoarthrit*" ) AND ALL ( "knee*" ) ) | 112937 |
| **#4** | TITLE-ABS-KEY ( "knee osteoarthritis" ) | 47468 |
| **#5** | TITLE-ABS-KEY ( "Osteoarthritis of Knee" ) | 392 |
| **#6** | TITLE-ABS-KEY ( "Osteoarthritis of Knees" ) | 392 |
| **#7** | TITLE-ABS-KEY ( "Knee OA" ) | 9454 |
| **#8** | TITLE-ABS-KEY ( "gonarthrosis" ) | 2154 |
| **#9** | #1 OR #2 OR #3 OR #4 OR #5 OR #6 OR #7 OR #8 | 113539 |
| **#10** | TITLE-ABS-KEY ("Proprioception") | 22963 |
| **#11** | TITLE-ABS-KEY ("Kinesthesis") | 3182 |
| **#12** | TITLE-ABS-KEY ("Postural Balance") | 23315 |
| **#13** | TITLE-ABS-KEY ("propriocep*") | 31680 |
| **#14** | TITLE-ABS-KEY ("kinesthes*") | 4340 |
| **#15** | TITLE-ABS-KEY ("position sense") | 2204 |
| **#16** | TITLE-ABS-KEY ("movement sense") | 159 |
| **#17** | TITLE-ABS-KEY ("velocity sense") | 19 |
| **#18** | TITLE-ABS-KEY ("force sense") | 228 |
| **#19** | TITLE-ABS-KEY ("sensorimotor training") | 420 |
| **#20** | TITLE-ABS-KEY ("balance training") | 3142 |
| **#21** | TITLE-ABS-KEY ("postural control") | 11997 |
| **#22** | TITLE-ABS-KEY ("neuromuscular training") | 907 |
| **#23** | TITLE-ABS-KEY ("stability training") | 411 |
| **#24** | TITLE-ABS-KEY ("kinesthetic training") | 34 |
| **#25** | TITLE-ABS-KEY ("core stability") | 1633 |
| **#26** | #10 OR #11 OR #12 OR #13 OR #14 OR #15 OR #16 OR #17 OR #18 OR #19 OR #20 OR #21 OR #22 OR #23 OR #24 OR #25 | 67040 |
| **#27** | TITLE-ABS-KEY ("Randomized Controlled Trial") | 1080778 |
| **#28** | TITLE-ABS-KEY ("Controlled Clinical Trial") | 464675 |
| **#29** | TITLE-ABS-KEY ("randomized") | 1473733 |
| **#30** | TITLE-ABS-KEY ("randomised") | 1473733 |
| **#31** | TITLE-ABS-KEY ("randomly") | 762332 |
| **#32** | TITLE-ABS-KEY ("trial") | 3105015 |
| **#33** | TITLE-ABS-KEY ("groups") | 9982328 |
| **#34** | #27 OR #28 OR #29 OR #30 OR #31 OR #32 OR #33 | 12610096 |
| **#35** | #9 AND #26 AND #34 | 775 |

**CINAHL complete N=174**

| CINAHL complete Search History | | |
| --- | --- | --- |
| **Search number** | **Query** | **Results** |
| **S1** | MH "osteoarthritis, knee" | 15365 |
| **S2** | MH "Osteoarthritis" AND TX "Knee" | 4788 |
| **S3** | TI "osteoarthrit*" OR AB "osteoarthrit*" | 38246 |
| **S4** | TX "knee*" | 153489 |
| **S5** | S3 AND S4 | 22150 |
| **S6** | TI "knee osteoarthritis" OR AB "knee osteoarthritis" | 10164 |
| **S7** | TI "Osteoarthritis of Knee" OR AB "Osteoarthritis of Knee" | 327 |
| **S8** | TI "Osteoarthritis of Knees" OR AB "Osteoarthritis of Knees" | 33 |
| **S9** | TI "Knee OA" OR AB "Knee OA" | 3954 |
| **S10** | TI "gonarthrosis" OR AB "gonarthrosis" | 208 |
| **S11** | S1 OR S2 OR S5 OR S6 OR S7 OR S8 OR S9 OR S10 | 27371 |
| **S12** | MH "Proprioception" | 3914 |
| **S13** | MH "Kinesthesis" | 787 |
| **S14** | TI "Postural Balance" OR AB "Postural Balance" | 734 |
| **S15** | TI "propriocep*" OR AB "propriocep*" | 4362 |
| **S16** | TI "kinesthes*" OR AB "kinesthes*" | 154 |
| **S17** | TI "position sense" OR AB "position sense" | 843 |
| **S18** | TI "movement sense" OR AB "movement sense" | 28 |
| **S19** | TI "velocity sense" OR AB "velocity sense" | 4 |
| **S20** | TI "force sense" OR AB "force sense" | 48 |
| **S21** | TI "sensorimotor training" OR AB "sensorimotor training" | 121 |
| **S22** | TI "balance training" OR AB "balance training" | 1399 |
| **S23** | TI "postural control" OR AB "postural control" | 3766 |
| **S24** | TI "neuromuscular training" OR AB "neuromuscular training" | 411 |
| **S25** | TI "stability training" OR AB "stability training" | 151 |
| **S26** | TI "kinesthetic training" OR AB "kinesthetic training" | 6 |
| **S27** | TI "core stability" OR AB "core stability" | 498 |
| **S28** | S12 OR S13 OR S14 OR S15 OR S16 OR S17 OR S18 OR S19 OR S20 OR S21 OR S22 OR S23 OR S24 OR S25 OR S26 OR S27 | 12606 |
| **S29** | PT "Randomized Controlled Trial" | 161218 |
| **S30** | TI "Controlled Clinical Trial" OR AB "Controlled Clinical Trial" | 7979 |
| **S31** | TI "randomized" OR AB "randomized" | 272222 |
| **S32** | TI "randomised" OR AB "randomised" | 59192 |
| **S33** | TI "randomly" OR AB "randomly" | 114276 |
| **S34** | TI "trial" OR AB "trial" | 299077 |
| **S35** | TI "groups" OR AB "groups" | 573957 |
| **S36** | S29 OR S30 OR S31 OR S32 OR S33 OR S34 OR S35 | 961352 |
| **S37** | S11 AND S28 AND S36 | 174 |

**WOS N=475**

| Web of Science Search History | | |
| --- | --- | --- |
| **Search number** | **Query** | **Results** |
| **#1** | TS=("osteoarthritis, knee") | 765 |
| **#2** | (TS=("Osteoarthritis")) AND ALL=("Knee") | 65971 |
| **#3** | (TS=("osteoarthrit*")) AND ALL=("knee*") | 67596 |
| **#4** | TS=("knee osteoarthritis") | 32922 |
| **#5** | TS=("Osteoarthritis of Knee") | 207 |
| **#6** | TS=("Osteoarthritis of Knees") | 15 |
| **#7** | TS=("Knee OA") | 8464 |
| **#8** | TS=("gonarthrosis") | 1055 |
| **#9** | #8 OR #7 OR #6 OR #5 OR #4 OR #3 OR #2 OR #1 | 68410 |
| **#10** | TS=("Proprioception") | 10129 |
| **#11** | TS=("Kinesthesis") | 123 |
| **#12** | TS=("Postural Balance") | 3730 |
| **#13** | TS=("propriocep*") | 17714 |
| **#14** | TS=("kinesthes*") | 791 |
| **#15** | TS=("position sense") | 2481 |
| **#16** | TS=("movement sense") | 77 |
| **#17** | TS=("velocity sense") | 9 |
| **#18** | TS=("force sense") | 190 |
| **#19** | TS=("sensorimotor training") | 357 |
| **#20** | TS=("balance training") | 2568 |
| **#21** | TS=("postural control") | 13257 |
| **#22** | TS=("neuromuscular training") | 789 |
| **#23** | TS=("stability training") | 277 |
| **#24** | TS=("kinesthetic training") | 15 |
| **#25** | TS=("core stability") | 1508 |
| **#26** | #10 OR #11 OR #12 OR #13 OR #14 OR #15 OR #16 OR #17 OR #18 OR #19 OR #20 OR #21 OR #22 OR #23 OR #24 OR #25 | 36757 |
| **#27** | TS=("Randomized Controlled Trial") | 216919 |
| **#28** | TS=("Controlled Clinical Trial") | 22178 |
| **#29** | TS=("randomized") | 949253 |
| **#30** | TS=("randomised") | 145373 |
| **#31** | TS=("randomly") | 502487 |
| **#32** | TS=("trial") | 1339923 |
| **#33** | TS=("groups") | 3175279 |
| **#34** | #27 OR #28 OR #29 OR #30 OR #31 OR #32 OR #33 | 4840864 |
| **#35** | #9 AND #26 AND #34 | 475 |

**EMBASE N=513**

| Embase Search History | | |
| --- | --- | --- |
| **Search number** | **Query** | **Results** |
| **#1** | 'knee osteoarthritis'/exp | 50172 |
| **#2** | 'osteoarthritis'/exp AND 'knee' | 75491 |
| **#3** | 'osteoarthrit*':ab,ti AND 'knee*' | 69101 |
| **#4** | 'knee osteoarthritis':ab,ti | 27883 |
| **#5** | 'osteoarthritis of knee':ab,ti | 425 |
| **#6** | 'osteoarthritis of knees':ab,ti | 37 |
| **#7** | 'knee oa':ab,ti | 15929 |
| **#8** | 'gonarthrosis':ab,ti | 1766 |
| **#9** | #1 OR #2 OR #3 OR #4 OR #5 OR #6 OR #7 OR #8 | 86135 |
| **#10** | 'proprioception'/exp | 17397 |
| **#11** | 'kinesthesia'/exp | 2600 |
| **#12** | 'body equilibrium'/exp | 25988 |
| **#13** | 'propriocep*':ab,ti | 19174 |
| **#14** | 'kinesthes*':ab,ti | 539 |
| **#15** | 'position sense':ab,ti | 2078 |
| **#16** | 'movement sense':ab,ti | 71 |
| **#17** | 'velocity sense':ab,ti | 10 |
| **#18** | 'force sense':ab,ti | 88 |
| **#19** | 'sensorimotor training':ab,ti | 364 |
| **#20** | 'balance training':ab,ti | 2921 |
| **#21** | 'postural control':ab,ti | 9572 |
| **#22** | 'neuromuscular training':ab,ti | 692 |
| **#23** | 'stability training':ab,ti | 300 |
| **#24** | 'kinesthetic training':ab,ti | 8 |
| **#25** | 'core stability':ab,ti | 934 |
| **#26** | #10 OR #11 OR #12 OR #13 OR #14 OR #15 OR #16 OR #17 OR #18 OR #19 OR #20 OR #21 OR #22 OR #23 OR #24 OR #25 | 60742 |
| **#27** | 'randomized controlled trial':ab,ti | 155484 |
| **#28** | 'controlled clinical trial':ab,ti | 27339 |
| **#29** | 'randomized':ab,ti | 1053737 |
| **#30** | 'randomised':ab,ti | 208935 |
| **#31** | 'randomly':ab,ti | 598524 |
| **#32** | 'trial':ab,ti | 1238891 |
| **#33** | 'groups':ab,ti | 3939070 |
| **#34** | #27 OR #28 OR #29 OR #30 OR #31 OR #32 OR #33 | 5507904 |
| **#35** | #9 AND #26 AND #34 | 513 |

**Cochrane Library N=511**

| Cochrane Library Search History |
| --- |

**ID Search Hits**

#1 MeSH descriptor: [Osteoarthritis, Knee] explode all trees 7083

#2 MeSH descriptor: [Osteoarthritis] explode all trees 11267

#3 ("Knee") 44762

#4 #2 AND #3 8430

#5 osteoarthrit*:ti,ab,kw 25989

#6 knee* 46115

#7 #5 AND #6 18981

#8 ("knee osteoarthritis"):ti,ab,kw 11178

#9 ("Osteoarthritis of Knee"):ti,ab,kw 1144

#10 ("Osteoarthritis of Knees"):ti,ab,kw 23

#11 ("Knee OA"):ti,ab,kw 3862

#12 ("gonarthrosis"):ti,ab,kw 588

#13 #1 OR #4 OR #7 OR #8 OR #9 OR #10 OR #11 OR #12 19172

#14 MeSH descriptor: [Proprioception] explode all trees 5279

#15 MeSH descriptor: [Kinesthesis] explode all trees 182

#16 MeSH descriptor: [Postural Balance] explode all trees 4511

#17 (propriocep*):ti,ab,kw 4273

#18 (kinesthes*):ti,ab,kw 284

#19 ("position sense"):ti,ab,kw 641

#20 ("movement sense"):ti,ab,kw 16

#21 ("velocity sense"):ti,ab,kw 1

#22 ("force sense"):ti,ab,kw 32

#23 ("sensorimotor training"):ti,ab,kw 245

#24 ("balance training"):ti,ab,kw 2487

#25 ("postural control"):ti,ab,kw 2195

#26 ("neuromuscular training"):ti,ab,kw 437

#27 ("stability training"):ti,ab,kw 304

#28 ("kinesthetic training"):ti,ab,kw 11

#29 ("core stability"):ti,ab,kw 781

#30 #14 OR #15 OR #16 OR #17 OR #18 OR #19 OR #20 OR #21 OR #22 OR #23 OR #24 OR #25 OR #26 OR #27 OR #28 OR #29 12813

#31 Randomized Controlled Trial:pt 294090

#32 Controlled Clinical Trial:pt 366986

#33 ("randomized"):ti,ab,kw 1229966

#34 ("randomised"):ti,ab,kw 1229966

#35 ("randomly"):ti,ab,kw 344959

#36 ("trial"):ti,ab,kw 1136684

#37 ("groups"):ti,ab,kw 654124

#38 #31 OR #32 OR #33 OR #34 OR #35 OR #36 OR #37 1767162

#39 #13 AND #30 AND #38 **511**

**S2: Risk of Bias 2 (ROB 2)**

***Domain 1: Bias arising from the randomization process***

**1.1 Is the allocation sequence random?**

- **Y (Yes): Computer-generated random sequence, random number table, coin tossing, shuffling cards/envelopes, throwing dice, drawing lots**
- **PY (Probably Yes): States "randomized" without further details but likely used proper method**
- **N (No): Quasi-random allocation method (e.g., by date of birth, clinic ID number, alternation)**
- **PN (Probably No): Suspicious randomization claims with evidence suggesting non-random allocation**
- **NI (No Information): Insufficient information to make judgment**

**1.2 Was the allocation sequence concealed?**

- **Y: Central allocation, sequentially numbered opaque sealed envelopes, other descriptions with convincing concealment**
- **PY: States that allocation was concealed with no further details**
- **N: Open random allocation schedule, assignments in unsealed/non-opaque envelopes, alternation or rotation**
- **PN: Small study with evidence suggesting potential allocation knowledge**
- **NI: Insufficient information to determine allocation concealment**

**1.3 Were there baseline imbalances suggesting problems with randomization?**

- **Y/PY: No notable baseline imbalances or imbalances consistent with chance**
- **N/PN: Significant baseline imbalances between intervention groups not explained by chance**
- **NI: No information about baseline characteristics or incomplete information**

**Risk judgment for Domain 1:**

- **Low risk: 1.1 = Y/PY AND 1.2 = Y/PY AND 1.3 = Y/PY/NI**
- **High risk: 1.1 = N/PN OR 1.2 = N/PN OR 1.3 = N/PN**
- **Some concerns: Any other combination**

***Domain 2 (Part 1): Bias due to deviations from intended interventions (2.1-2.5)***

**2.1 Were participants aware of their assigned intervention?**

- **Y: No blinding and nature of intervention makes awareness inevitable**
- **PY: No blinding but likelihood of awareness**
- **N: Blinding reported and unlikely to be broken**
- **PN: Blinding reported but possibility it could be broken**
- **NI: No information on blinding**

**2.2 Were personnel/implementers aware of assigned intervention?**

- **Y: No blinding of intervention staff**
- **PY: No report of blinding but likely aware**
- **N: Effective blinding of intervention staff**
- **PN: Blinding stated but possibly broken**
- **NI: No information provided**

**2.3 Were there deviations from the intended intervention due to study context?**

- **Y/PY: Important deviations from intended intervention**
- **N/PN: No or minimal deviations from intended intervention**
- **NI: Insufficient information to assess**

**2.4 Did deviations affect the outcome?**

- **Y/PY: Deviations likely to impact outcome**
- **N/PN: Deviations unlikely to impact outcome**
- **NI: Insufficient information**

**2.5 Were deviations balanced between groups?**

- **Y/PY: Deviations balanced between groups**
- **N/PN/NI: Deviations not balanced or insufficient information**

**Risk judgment for Domain 2 (Part 1):**

- **Low risk: (All 2.1-2.2 = N/PN) OR (2.3 = N/PN) OR (2.4 = N/PN) OR (2.5 = Y/PY)**
- **High risk: (2.3 = Y/PY AND 2.4 = Y/PY/NI AND 2.5 = N/PN/NI)**
- **Some concerns: Any other combination**

***Domain 2 (Part 2): Bias due to deviations from intended interventions (2.6-2.7)***

**2.6 Was an appropriate analysis used to estimate the effect of assignment to intervention?**

- **Y/PY: Intention-to-treat (ITT) analysis or modified ITT with minimal missing data**
- **N/PN/NI: As-treated analysis, per-protocol analysis, or significant missing data compromising ITT**

**2.7 Was there potential for substantial impact of failure to analyze according to assigned intervention?**

- **Y/PY: Substantial proportion of participants did not receive assigned intervention**
- **N/PN: Most participants received assigned intervention or impact of deviation minimal**
- **NI: No information provided on intervention adherence**

**Risk judgment for Domain 2 (Part 2):**

- **Low risk: 2.6 = Y/PY**
- **High risk: (2.6 = N/PN/NI AND 2.7 = Y/PY/NI)**
- **Some concerns: Any other combination**

**Overall judgment for Domain 2:**

- **Low risk: Both Part 1 and Part 2 = Low risk**
- **High risk: Either Part 1 OR Part 2 = High risk**
- **Some concerns: Any other combination**

***Domain 3: Bias due to missing outcome data***

**3.1 Were outcome data available for all randomized participants?**

- **Y/PY: No missing data or minimal missing data (<5%)**
- **N/PN/NI: Notable missing data (≥5%) or insufficient information**

**3.2 If No/PN/NI to 3.1: Are the proportions of missing outcome data and reasons similar across groups?**

- **Y/PY: Similar proportions and reasons for missing data across groups**
- **N/PN: Different proportions or reasons for missing data across groups**

**3.3 If No/PN/NI to 3.1: Is there evidence that results were robust to the presence of missing data?**

- **Y/PY: Appropriate methods for handling missing data (multiple imputation, sensitivity analyses)**
- **N/PN: Inappropriate handling of missing data or methods that may bias results**
- **NI: No information on how missing data were addressed**

**3.4 If No/PN/NI to 3.3: Could missingness depend on true value of outcome?**

- **Y/PY/NI: Missing data likely dependent on true outcome value**
- **N/PN: Missing data likely independent of true outcome value**

**Risk judgment for Domain 3:**

- **Low risk: 3.1 = Y/PY OR (3.2 = Y/PY AND 3.3 = Y/PY)**
- **High risk: (3.2 = N/PN OR 3.3 = N/PN) AND 3.4 = Y/PY/NI**
- **Some concerns: Any other combination**

***Domain 4: Bias in measurement of the outcome***

**4.1 Was the method of measuring the outcome inappropriate?**

- **Y/PY: Measurement methods inappropriate or could lead to bias**
- **N/PN: Measurement methods appropriate and validated**
- **NI: Insufficient information on measurement methods**

**4.2 Could measurement of outcome differ between intervention groups?**

- **Y/PY: Outcome assessors aware of intervention status and potential for bias**
- **N/PN: Outcome measurement objective or assessors blinded to intervention**
- **NI: No information on whether outcome assessors were aware of intervention**

**4.3 If N/PN/NI to 4.2: Were outcome assessors aware of the intervention received?**

- **Y/PY/NI: Outcome assessors aware of intervention or insufficient information**
- **N/PN: Effective blinding of outcome assessors**

**4.4 If Y/PY/NI to 4.3: Could assessment of the outcome have been influenced by knowledge of intervention received?**

- **Y/PY/NI: Subjective outcomes with potential for bias in assessment**
- **N/PN: Objective outcomes unlikely to be influenced by knowledge of intervention**

**4.5 If Y/PY/NI to 4.4: Is it likely that assessment of the outcome was influenced by knowledge of intervention received?**

- **Y/PY/NI: Evidence suggests assessment was influenced by knowledge of intervention**
- **N/PN: No evidence that assessment was influenced by knowledge of intervention**

**Risk judgment for Domain 4:**

- **Low risk: (4.1 = N/PN AND 4.2 = N/PN) OR (4.3 = N/PN) OR (4.4 = N/PN) OR (4.5 = N/PN)**
- **High risk: (4.1 = Y/PY) OR (4.2 = Y/PY) OR (4.5 = Y/PY/NI)**
- **Some concerns: Any other combination**

***Domain 5: Bias in selection of the reported result***

**5.1 Were the data that produced this result analyzed in accordance with a pre-specified analysis plan?**

- **Y/PY: Analysis consistent with pre-specified plan in protocol/registry**
- **N/PN/NI: Analysis not specified in advance or insufficient information**

**5.2 & 5.3 Was there selective reporting from multiple outcome measurements or analyses?**

- **N/PN: All relevant outcomes/analyses reported as specified**
- **Y/PY: Evidence of selective reporting from multiple analyses or outcomes**
- **NI: Insufficient information to determine selective reporting**

**Risk judgment for Domain 5:**

- **Low risk: All 5.1-5.3 = N/PN**
- **High risk: Any 5.1-5.3 = Y/PY**
- **Some concerns: Any other combination including NI responses**

**Overall Risk of Bias Judgment**

**Low risk: All domains judged to be at low risk.**

**Some concerns: At least one domain judged to have some concerns, but no domain at high risk.**

**High risk: At least one domain judged to be at high risk, or multiple domains with some concerns that substantially lower confidence in the result.**

*Abbreviations used in the assessment:* Y = Yes, PY = Probably Yes, NI = No Information, PN = Probably No, N = No.

**S3: Detail Data of Sensitivity Analysis**

**Table 1. Sensitivity Analysis for TUG**

| **Study omitted** | **Estimate** | **[95% Conf. Interval]** |
| --- | --- | --- |
| Bhaskar et al. (2019) | -1.4540001 | (-1.9364673, -0.97153276) |
| Gomiero et al. (2018) | -1.6862273 | (-2.1738253, -1.1986292) |
| Hale et al. (2012) | -1.5458418 | (-1.9921132, -1.0995704) |
| Jahanjoo et al. (2019) | -1.3707567 | (-1.8937123, -0.84780104) |
| Joshi et al. (2023) | -1.5691303 | (-2.0701496, -1.0681111) |
| Sharma et al. (2018) | -1.5780285 | (-2.051797, -1.10426) |
| Tudpor et al. (2021) | -1.488841 | (-1.9346585, -1.0430236) |
| **Combined** | **-1.5296948** | **(-1.9721406, -1.0872491)** |

**Table 2. Sensitivity Analysis for WOMAC-total**

| **Study omitted** | **Estimate** | **[95% Conf. Interval]** |
| --- | --- | --- |
| Duman et al. (2012) | -3.5783308 | (-5.4112522, -1.7454095) |
| Fitzgerald et al. (2011) | -3.43506 | (-5.3723789, -1.4977411) |
| Gomiero et al. (2018) | -3.6198176 | (-5.4550526, -1.7845827) |
| Hale et al. (2012) | -3.6292778 | (-5.4856047, -1.7729509) |
| Jahanjoo et al. (2019) | -4.2909505 | (-6.2695501, -2.3123509) |
| Joshi et al. (2023) | -2.9581432 | (-4.85733, -1.0589564) |
| Kuş et al. (2023) | -3.6154855 | (-5.4458269, -1.7851441) |
| Rashid et al. (2019) | -2.8110296 | (-4.7667075, -0.85535176) |
| Rathwa et al. (2019) | -2.3160457 | (-4.2480363, -0.38405505) |
| Rogers et al.-a (2012) | -3.4422029 | (-5.241184, -1.6432218) |
| Rogers et al.-b (2012) | -3.3879281 | (-5.1873282, -1.5885279) |
| Sharma et al. (2018) | -3.316518 | (-5.1445804, -1.4884556) |
| **Combined** | **-3.3718733** | **(-5.1629906, -1.580756)** |

**Table 3. Sensitivity Analysis for WOMAC-pain**

| **Study omitted** | **Estimate** | **[95% Conf. Interval]** |
| --- | --- | --- |
| Arif et al. (2022) | -0.62715822 | (-1.4983965, 0.24408003) |
| Duman et al. (2012) | -1.215071 | (-2.7288494, 0.29870736) |
| Hale et al. (2012) | -1.3331749 | (-2.7875467, 0.12119692) |
| Jahanjoo et al. (2019) | -1.1990998 | (-2.7708647, 0.37266504) |
| Lin et al.-a (2009) | -0.88790275 | (-2.3594044, 0.58359888) |
| Lin et al.-b (2009) | -1.2672604 | (-2.7718217, 0.2373009) |
| Ojoawo et al. (2016) | -0.93765904 | (-2.4041259, 0.52880779) |
| Rogers et al.-a (2012) | -1.2288076 | (-2.6488744, 0.19125911) |
| Rogers et al.-b (2012) | -1.2426103 | (-2.663468, 0.17824744) |
| Tsauo et al. (2008) | -1.2460445 | (-2.7923096, 0.30022063) |
| **Combined** | **-1.1229026** | **(-2.4883942, 0.24258901)** |

**Table 4. Sensitivity Analysis for WOMAC-stiffness**

| **Study omitted** | **Estimate** | **[95% Conf. Interval]** |
| --- | --- | --- |
| Duman et al. (2012) | 0.03482423 | (-0.3389999, 0.40864835) |
| Hale et al. (2012) | -0.03687719 | (-0.38604804, 0.31229366) |
| Jahanjoo et al. (2019) | -0.04021458 | (-0.39111076, 0.3106816) |
| Ojoawo et al. (2016) | 0.155778 | (-0.23014456, 0.54170055) |
| Rogers et al.-a (2012) | -0.00196912 | (-0.33357473, 0.32963649) |
| Rogers et al.-b (2012) | -0.03483599 | (-0.36832704, 0.29865507) |
| Tsauo et al. (2008) | 0.008049 | (-0.33859366, 0.35469165) |
| **Combined** | **0.00710069** | **(-0.31848204, 0.33268341)** |

**Table 5. Sensitivity Analysis for WOMAC-function**

| **Study omitted** | **Estimate** | **[95% Conf. Interval]** |
| --- | --- | --- |
| Arif et al. (2022) | -1.3107033 | (-4.1661453, 1.5447386) |
| Duman et al. (2012) | -3.7977032 | (-8.7683829, 1.1729766) |
| Fitzgerald et al. (2011) | -3.425794 | (-8.9069575, 2.0553695) |
| Hale et al. (2012) | -3.6053232 | (-8.7870748, 1.5764285) |
| Jahanjoo et al. (2019) | -3.8262098 | (-9.0876212, 1.4352017) |
| Lin et al.-a (2009) | -2.6133651 | (-7.4995722, 2.2728419) |
| Lin et al.-b (2009) | -4.2192009 | (-9.09404, 0.6556382) |
| Ojoawo et al. (2016) | -2.9006726 | (-7.9208341, 2.119489) |
| Rogers et al.-a (2012) | -3.777404 | (-8.5893308, 1.0345228) |
| Rogers et al.-b (2012) | -3.4082856 | (-8.2502245, 1.4336532) |
| Tsauo et al. (2008) | -3.907255 | (-8.9211205, 1.1066105) |
| **Combined** | **-3.3520121** | **(-7.988441, 1.2844168)** |

**Table 6. Sensitivity Analysis for NRS**

| **Study omitted** | **Estimate** | **[95% Conf. Interval]** |
| --- | --- | --- |
| Arif et al. (2022) | -0.78498532 | (-1.0871345, -0.48283609) |
| Fitzgerald et al. (2011) | -0.8986583 | (-1.2188917, -0.57842486) |
| Joshi et al. (2023) | -0.87441115 | (-1.2312839, -0.51753838) |
| Kumar et al. (2013) | -0.95892561 | (-1.3600827, -0.55776848) |
| Tudpor et al. (2021) | -0.81011565 | (-1.1106875, -0.50954381) |
| **Combined** | **-0.85413185** | **(-1.1495308, -0.5587329)** |

**Table 7. Sensitivity Analysis for VAS**

| **Study omitted** | **Estimate** | **[95% Conf. Interval]** |
| --- | --- | --- |
| Apparao et al. (2017) | -0.19300415 | (-0.35506351, -0.03094478) |
| Gomiero et al. (2018) | -0.23169885 | (-0.38379915, -0.07959854) |
| Hussein et al. (2010) | -0.21710766 | (-0.37073803, -0.0634773) |
| Jahanjoo et al. (2019) | -0.20765018 | (-0.36329073, -0.05200964) |
| Oh et al. (2020) | -0.21164045 | (-0.36366732, -0.05961357) |
| Sharma et al. (2018) | -0.25293006 | (-0.40726971, -0.0985904) |
| Sobhani et al. (2024) | -0.21803026 | (-0.48928459, 0.05322408) |
| **Combined** | **-0.2193911** | **(-0.37020675, -0.06857544)** |

**S4: Figure Captions (supplementary figures)**

Figure 1. Sensitivity analysis forest plot and Egger's test of TUG, (A) Sensitivity analysis forest plot, (B) Egger's test

Figure 2. Sensitivity analysis forest plot and Egger's test of WOMAC-total, (A) Sensitivity analysis forest plot, (B) Egger's test

Figure 3. Sensitivity analysis forest plot and Egger's test of WOMAC-pain, (A) Sensitivity analysis forest plot, (B) Egger's test

Figure 4. Sensitivity analysis forest plot and Egger's test of WOMAC-stiffness, (A) Sensitivity analysis forest plot, (B) Egger's test

Figure 5. Sensitivity analysis forest plot and Egger's test of WOMAC-function, (A) Sensitivity analysis forest plot, (B) Egger's test

Figure 6. Sensitivity analysis forest plot and Egger's test of NRS, (A) Sensitivity analysis forest plot, (B) Egger's test

Figure 7. Sensitivity analysis forest plot and Egger's test of VAS, (A) Sensitivity analysis forest plot, (B) Egger's test

Figure 8. Forest plot of intervention type subgroup analysis of WOMAC-pain and WOMAC-function, (A) WOMAC-pain, (B) WOMAC-function

Figure 9. Forest plot of study region subgroup analysis of WOMAC-pain and WOMAC-function, (A) WOMAC-pain, (B) WOMAC-function
